# An IGF-1R-mTORC1-SRPK2 signaling Axis contributes to FASN regulation in breast cancer

**DOI:** 10.1186/s12885-022-10062-z

**Published:** 2022-09-12

**Authors:** Bryan McClellan, Paul Gries, Brittany Harlow, Stefano Tiziani, Christopher Jolly, Linda deGraffenried

**Affiliations:** 1 Department of Nutritional Sciences, College of Natural Sciences, The University of Texas at Austin, Austin, TX 78712 USA; 2 Department of Pediatrics, Dell Medical School, The University of Texas at Austin, Austin, TX 78723 USA; 3 Department of Oncology, Dell Medical School, Livestrong Cancer Institutes, The University of Texas at Austin, 1400 Barbara Jordan Blvd, Austin, TX 78723 USA

**Keywords:** RTK signaling, RNA metabolism, Cancer metabolism, Breast Cancer, Fatty acid synthase

## Abstract

**Background:**

Fatty acid synthase (FASN) expression is associated with a more aggressive breast cancer phenotype and is regulated downstream of receptor tyrosine kinase (RTK) signaling pathways. Recently, post transcriptional regulation of lipogenic transcripts have been demonstrated as being mediated downstream of serine-arginine rich protein kinase 2 (SRPK2), which acts to phosphorylate serine-arginine rich splicing factors (SRSFs), resulting in RNA binding and various RNA regulatory processes. Though post-transcriptional regulation of FASN has been studied previously, the upstream mediators of these pathways have not been elucidated.

**Methods:**

Western blotting and RT-qPCR were utilized to demonstrate alterations in FASN and mRNA expression upon modulation of the IGF-1-mTORC1-SRPK2 pathway by small molecule inhibitors or RNAi mediated silencing. RNA stability was accessed by using the transcriptional inhibitor actinomycin-D followed by RT-qPCR. Further, we employed RNA-immunoprecipitation to demonstrate the direct binding of SRSF-1 to FASN transcripts.

**Results:**

In the current study, we demonstrated an IGF-1 induced increase in FASN mRNA and protein expression that was attenuated by mTORC1 inhibition. This mTORC1 inhibition also resulted in decreases in total and nuclear p-SRPK2 in response to IGF-1 exposure. Upon SRPK2 knockdown and inhibition, we observed a decrease in FASN protein and mRNA stability, respectively, in response to IGF-1 exposure that was specific to triple negative and HER2+ breast cancer cell lines. As we explored further, IGF-1 exposure resulted in an altered localization of eGFP expressed SRSF-1, pEGFP-SRSF-1 that was rescued upon both SRPK2 knockdown and mTORC1 inhibition. Further, we observed an increase binding of SRSF-1 to FASN RNA upon IGF-1 exposure, which was abrogated by SRPK2 knockdown.

**Conclusion:**

These current findings establish a potential IGF-1-mTORC1-SRPK2-FASN axis in breast cancer, which could be a potential therapeutic target for cancers that overexpress FASN and components of the IGF-1R pathway.

**Supplementary Information:**

The online version contains supplementary material available at 10.1186/s12885-022-10062-z.

## Background

Altered cellular metabolism is an established hallmark in cancer and one of the most active areas in therapeutic research today [1–3]. Non-proliferative tissues often obtain adequate fatty acids from diet; however, cancer cells often display aberrant de novo fatty acid synthesis to supply ample substrates for post-translational lipidations, phospholipids, and energy [3]. The de novo fatty acid synthesis pathway is mediated through several enzymatic steps beginning with the synthesis of palmitate from various substrates including glucose and glutamine by the fatty acid synthase enzyme (FASN) [3]. Moreover, FASN is overexpressed in numerous solid tumors and its expression is associated with a more aggressive disease [4–7]. FASN expression and subsequent palmitate production can lead to cancer progression through multiple mechanisms including providing substrates for protein palmitoylation, lipid droplet formation, phospholipid synthesis, or β-oxidation [3]. Additionally, palmitate can undergo further desaturation and/or elongation by sterol-CoA desaturase (SCD) and elongases (ELOVL), respectively, to be incorporated into phospholipids or lipid droplets [3].

Recently, there has been elucidation of post-transcriptional regulation of lipogenic genes through various metabolic signaling networks including the Akt-mTORC1 and hexosamine biosynthetic pathway (HBP) [8, 9]. Both of which seem to act in concert to promote the nuclear localization and phosphorylation of serine/arginine rich protein kinase 2 (SRPK2). SRPKs regulate many post-transcriptional processes, including mRNA nuclear export, mRNA decay, and pre-mRNA splicing through the phosphorylation serine-arginine rich (SR) proteins [8, 10]. Pre-mRNA splicing is mediated through the recruitment of the spliceosome, a complex made up of small nuclear ribonuclear proteins (snRNP) [11]. Spliceosome recruitment to nascent transcripts occurs through non-SnRNP splicing factors, which can be broadly categorized as either heterogenous nuclear ribonuclear proteins (hnRNPs) or serine/arginine rich (SR) proteins [12]. SR proteins are a family of RNA binding proteins that contain regions rich in arginine-serine dipeptides (SR domain) as well as RNA recognition motifs (RRMs), which mediate their phosphorylation and ESE RNA binding, respectively [11, 12]. Intron retention (IR), in which non-coding intron transcripts are retained in the fully processed and spliced mRNA transcript, is a common form of alternative splicing [13, 14]. IR transcripts are either spliced and translated as variants or they are degraded in the cytoplasm by the nonsense mediated decay (NMD) pathway [14]. Previous work has identified pre-mRNA splicing by SR protein recruitment of the snRNP, U1-70K, in mediating lipogenic mRNA stability [8].

Growth factors, such as EGF, FGF, IGF-1, allow for contextual cues from the extracellular environment to dictate cellular and molecular outcomes [15–17]. Although intricate studies have illustrated the roles of growth factors in controlling pre-mRNA splicing and RNA processing, the precise role of growth factor signaling and lipogenic regulation through post-transcriptional processing warrants further elucidation. The Insulin-like growth factor receptor (IGF-1R), is highly expressed in breast cancers, such as estrogen receptor positive (ER+) and triple negative (TNBC) and its expression is associated with a worse clinical prognosis [18, 19] Upon dimerization and activation by IGF-1, IGF-1R activates many downstream signaling cascades including the Akt-mTORC1 pathway that controls many metabolic processes [20]. Thus, the present study identifies the IGF-1-IGF-1R-mTOR axis as an integral signaling axis in the regulation of FASN in breast cancer. More specifically, IGF-1 induces enhanced FASN mRNA stability through the SRPK2 and SRSF-1 proteins. These results offer an enhanced insight into a novel metabolic regulatory pathway as well as offer potential therapeutic targets for breast cancers that both overexpress IGF-1R and display accentuated de novo fatty acid synthesis.

## Methods

### Cell lines

Human breast cancer cells MCF-7, MDA-MB-231 and non-transformed mammary epithelial cells, MCF-10A, were purchased from the American Type Culture Collection (ATCC, Manassas, VA). MDA-MB-453, SUM-159, BT-549, and SKBR3 cells were a kind gift from Dr. Ratna Vadlamudi at the University of Texas Health in San Antonio. All cells were maintained in Roswell Park Memorial Institute (RPMI) medium supplemented with 10% FBS and 1% penicillin/streptomycin. SUM-159 cell medium also contained hydrocortisone (1μg/mL). MCF-7 cells were cultured in Eagles Minimum Essential Medium supplemented with human recombinant insulin (.05 mg/ml), 10% fetal bovine serum (FBS), and 1% penicillin/streptomycin. MDA-MB-231 cells were cultured in Dulbecco’s Modified Eagles’ Medium (DMEM) supplemented with 10% FBS, and 1% penicillin/streptomycin. MCF-10A cells were cultured in DMEM supplemented with 10% FBS, EGF (20 ng/ml), hydrocortisone (.5 mg/ml), insulin (10 μg/ml), cholera toxin (100 ng/ml), and penicillin/streptomycin (1%). All cells were kept at 37 ° C and supplemented with 5% CO_2_.

### SiRNA transfections

MDA-MB-231, MDA-MB-453, SUM-159, BT-549, and SKBR3 cells were transfected with lipofectamine RNAi Max (Thermo Fisher, #13778075) transfection reagent according to manufacturer’s protocol. IGF-1R siRNA (Cell Signaling Technology (CST), #6610S) or Control siRNA (CST, #6568) were diluted in OPTIMEM reduced serum medium (Thermo Fisher, #31985062) at a concentration of 100 nM and complexed with lipofectamine RNAi reagent. MCF-7 cells were transfected with FUGENE transfection reagent (Promega, #E2311) according to manufacturer’s protocol with either 100 nM of IGF-1R or control siRNA. SiRNA and FUGENE reagents were mixed at a ratio of 5:1 FUGENE: siRNA and transfected in antibiotic free full growth media for 12 hours. MCF-7 cells were then serum starved for 12 hours and exposed to (100 ng/mL) IGF-1 for 24 hours. The total transfection time was 48 hours. For SRPK2 (Millipore Sigma, #SASI_Hs01_00057789), SRSF-1 (Ambion, #n546318), and negative control (Millipore Sigma, #SIC003) siRNAs, both MCF-7 and MDA-MB-231 cells were transfected with lipofectamine RNAi reagent (Thermo Fisher, #13778075). Briefly, siRNAs were diluted in OPTIMEM along with lipofectamine reagent and allowed to complex for 20 minutes at room temperature. The diluted mixture was then added to the cells and allowed to incubate at 37 °C for 6 hours. After 6 hours the medium was replaced with normal growth medium until further treatment or serum starving. The total transfection time was 48 hours.

### Plasmid transfections

The pEGFP-SF2 (pEGFP-SRSF-1) plasmid vector was a kind gift from Tom Misteli (Addgene, #17990). pEGFP-SRSF-1 was transfected into MCF-7 and MDA-MB-231 cells using lipofectamine 2000 reagent (Thermo Fisher, #11668027) according to manufacturer’s instructions. Briefly, DNA complexes and lipofectamine were mixed at a 1μg:1ul ratio in OPTIMEM media and incubated at room temperature for 20 minutes before being added to cells for 3-6 hours. For the co-transfection experiment: 10 nM of control or SRPK2 siRNA were added along with .2μg of pEGFP-SRSF-1 in OPTIMEM. Lipofectamine 2000 reagent was then added and allowed to complex for 20 minutes before being added to the cells for 3 hours. Full growth media was then added until subsequent treatments and/or serum starving. All transfections were carried out for 48 hours.

### RT-qPCR

RNA was extracted from MDA-MB-231, MCF-7, MCF-10A using Trizol reagent according to the manufacturer’s instructions. 2000 ng of RNA was subjected to reverse transcription using Moloney Murine leukemia virus (MLMTV)- reverse transcriptase according to manufacturer’s protocol (Invitrogen, #28025-013), random hexamers (Thermo Scientific, #00986258), RT buffer (Applied Biosystems, #4319981) and dNTPs (Fisher Bioreagents, #BP2564-1). The resulting cDNA was diluted 5-fold and subjected to qPCR analysis with Power UP SYBER green master mix (Thermo Fisher, #A25741) and designated primers (sequences in Table S1), Ct values were standardized to actin and made relative to the negative control (dCT) as a fold change (2-^ddCT).

### Intron retention PCR analysis

MDA-MB-231 cells were transfected as described in previous sections with either control or SRPK2 siRNA for 24 hours. After 24 hours, cells were serum starved for 16 hours prior to the addition of IGF-1 or full growth medium alone for an additional 24 hours. After 48 total hours, RNA was extracted, diluted to 2000 ng, and subjected to reverse transcription as described under RT-qPCR section. cDNA was then subjected to qPCR with SYBR green master mix according to manufacturer’s instructions. FASN intron 4 (intron included) and exon 4 (intron excluded) primers (sequences in Table S1) were used to amplify the cDNA. Intron 4 Ct values were normalized to Exon 4 and expressed as a relative fold change to the (−) IGF-1 condition by the 2^-ddCT method. The mean of 3 independent trials (*n =* 3) ± S.E.M. were expressed as a relative fold change to no-IGF-1 exposure.

### mRNA stability

MCF-10A, MCF-7, and MDA-MB-231 were pretreated with or without SRPN-340 (3.5 μM) in serum free media for 18 hours followed by (5 μg/mL) actinomycin-D treatment for 0, 3,5 hours in full growth media. RNA was extracted and reverse transcribed using the same protocol as the RT- qPCR gene expression listed previously. All genes were normalized to the 0 actinomycin-D condition for either Vehicle (DMSO) or SRN-340 treated.

### Western blotting

Cells were treated and lysed in 6-well dishes using 100 uL of cell lysis buffer (1% SDS, 1 mM EDTA, 10 mM Tris-HCL (pH 8.0)) supplemented with Halt phosphatase and protease inhibitor cocktail kit (Thermo Fisher #787429). Lysates were sonicated to shear DNA 3 × 10 seconds with one minute rest on ice using a Misonix ultrasonic liquid processor. Lysates were then centrifuged 10,000 rpm at 4̊C for 10 minutes. The resulting supernatant was collected and 10 uL subjected to Pierce BCA protein assay (Thermo Fisher #23227). 50 μg of protein were solubilized in 4x loading buffer (8% SDS, 250 mM Tris-Hcl (PH 6.8), 20% β-mercapthoethanol, 40% glycerol, and .2% bromophenol blue) and heated to 95̊ c for 5 minutes. Lysates were resolved on an 8% SDS-PAGE and transferred to a nitrocellulose membrane for one hour. Membranes were cut and blocked in 5% milk in TBST for one hour at room temperature and washed with TBST for 5 minutes. Primary antibodies (Table S1.) in dilution buffer (SRPK 1:300, GAPDH 1:300, S6K 1:300, p-S6K 1:300, all other primary antibodies were 1:1000) were incubated at 4̊C overnight. Membranes were washed the following day with TBST once for 15 minutes followed by an additional 5 minutes wash. Secondary antibodies anti-rabbit HRP-linked IgG (CST, #7074) (1:3000) or anti-mouse HRP-linked IgG (CST, #7076) (1:5000) were diluted in 5% milk and added to the membranes for 1 hour at room temperature. Membranes were washed in TBST 3 times for 5 minutes and incubated with West ATTO chemiluminescent substrate (Thermo Fisher, #A38554) prior to being visualized with a Syngene imaging system using the gene snap technology program.

### Immunofluorescence and fluorescence microscopy

MDA-MB-231 and MCF-7 cells were grown on 16-well chamber slides (Lab-Tek, #78599) and serum starved with rapamycin or DMSO for 16 hours followed by exposure to either full growth media alone or full growth media and IGF-1 (100 ng/mL) for 0, 2, 6, 12, or 18 hours. For SRSF-1 localization, cells were transfected with either pEGFP-SRSF-1 alone or co-transfected with SRPK2 siRNA or control. Transfections were followed by serum starvation with DMSO or rapamycin (100 nM) for 16 hours and IGF-1 exposure for 0, 3, and 6 hours in full growth medium. Following respective treatments, media was removed, and cells were fixed with 3.7% paraformaldehyde in PBS for 20 minutes at room temperature. Slides were then blocked for one hour in blocking buffer (5% BSA, PBS, and .3% Triton-X-100) at room temperature. Chambers were then removed, and slides washed for 5 minutes in 1XPBS. SRPK2 (BD Biosciences, #61118) was diluted in blocking buffer (1:100) and added to the slides. Slides were incubated overnight (16 hours) at 4 ° C. The following day, coverslips were removed, and slides were washed three times in 1xPBS for five minutes each. Secondary antibodies, Cy3 anti-mouse IgG conjugate (Jackson laboratories, ##711-095-152) (1:1200) were diluted in the same blocking buffer and added to the slides for two hours at room temperature while protected from light. Coverslips were removed and slides were washed again three times for five minutes each in 1xPBS. Slides were then allowed to dry and mounted with Prolong Gold Antifade reagent (Invitrogen, #P36966) containing DAPI. Mounted slides were sealed with nail polish and allowed to dry for 24 hours at room temperature protected from light. Images were obtained using a Leica Confocal microscope. Images were processed using Adobe photoshop and NIH Image J software. Quantification of nuclear localization was performed using a FIJI (image J) plugin as described in [21].

### Cell fractionation

MDA-MB- 231 and MCF-7 cells were grown in 100 mm dishes until confluency and serum starved overnight (16 hours) with either DMSO or rapamycin (100 nM) followed by exposure to IGF-1 (100 ng/m) in full growth media with 10% FBS for 6 hours. Cell media was removed, and dishes were washed twice with cold 1XPBS after which .3 mL of lysis buffer (.33 M sucrose, 10 mM HEPES, PH 7.4, 1 mM MgCl_2,_ 0.1% Triton-X-100) was added to the dish. Cells were then scraped and transferred to chilled 1.5 mL tubes and incubated for an additional 15 minutes on ice and centrifuged for 10 minutes, 10,000 rpm at 4 ° C. Cytosolic fractions were removed and kept on ice. The resulting pellets were washed twice in lysis buffer and suspended in .1 mL of nuclear lysis buffer (.45 M NaCl, 10 mM HEPES, PH 7.4, 1 mM MgCl_2_) for 30 minutes on ice. Lysates were spun at 14,000 rpm for 5 minutes prior to be harvested. Both cytosolic and nuclear fractions were subjected to a pierce BCA protein assay to determine protein concentration. 50 μg of lysate was solubilized in 4x SDS loading buffer and resolved on an 8% SDS-PAGE and transferred to a nitrocellulose membrane for 1 hour. The remaining western blotting was identical to the protocol listed under western blotting section. Bands were quantified in NIH Image J software and standardized to their loading control. Cytosolic p-SRPK2 band intensity was standardized to GAPDH, and nuclear p-SRPK was standardized to HDAC1. All experiments were performed in biological triplicate.

### Sample preparation for GC-MS lipid analysis

MDA-MB-231 and MCF-7 cells were gown in a T75 flask for 24 hours prior to siRNA transfection with either 10 nM control siRNA (Millipore Sigma, #SIC003) or 10 nM of SRPK2 siRNA (Millipore Sigma, # SASI_Hs01_00057789) in OPTIMEM for at least 6 hours with lipofectamine RNAi transfection reagent (thermo fisher, #13778075) according to manufacturer’s instructions. After transfection, the medium was replaced full growth medium + 10% FBS until the following day. After 24 hours, glucose-free RPMI medium containing 10% charcoal stripped FBS, 4.5 g/L of [U-C^13^] glucose isotope (Cambridge isotopes, #.110187-42-3), and 100 ng/mL IGF-1 was used to treat the cells for an additional 48 hours. After the 48-hour treatment, cells were suspended by trypsinization, spun down, and washed with cold 1xPBS two times. The pellets were then transferred to 1.5 mL tubes and spun again at max speed. The supernatant was discarded, and pellets were flash frozen in liquid nitrogen and stored at − 80 ° C until further analysis.

### GC-MS fatty acid analysis

Lipid metabolites were isolated through a modified Folch extraction and analyzed by GC-MS [22]. Cells and media samples were combined with 800 μL HPLC Grade isooctane (Thermo Fisher Scientific, Waltham, MA) 200 μL of HPLC Grade methanol, 100 μL of 0.9% saline, and 20 μL of 1 N HCl. Additionally, 10 μL of the antioxidant butylated hydroxytoluene dissolved in ethanol at 200 μg/mL and 20 μL of the internal standard, deuterated heptadecanoic acid (C17:0) (Cayman Chemicals; Ann Arbor, Michigan, MI) were added for a final concentration of 20 μg/mL.

This mixture was vortexed and centrifuged in a Beckman Coulter Allegra X-15r Centrifuge (Brea, CA, USA) for 20 minutes at 4 °C for polar-apolar phase separation. The top apolar phase was transferred to a new tube and dried at 4 °C using refrigerated Labconco CentriVap Concentrator attached to − 84 °C CentriVap Cold Trap (Kansas City, MO, USA). Metabolites were derivatized by combining 30 μL of trimethyl sulfonium hydroxide (Sigma-Aldrich) (20 mg/mL) and 60ul of methyl tert-butyl ether (Sigma- Aldrich) in an automated manner as described in previous publication [23] A small volume (3 μL) of derivatized samples were injected via spitless injection into a Thermo Scientific Thermo Scientific Trace 1310 gas chromatograph loaded with a Phenomenex Zebron ZB- (Torrance, CA, USA) 1 ms fused silica capillary column (length = 30 m, I.D. = 0.25 mm, film = 0.25 μm), which was connected to a Thermo ISQ (Thermo Fisher Scientific) single quadrupole mass spectrometer. Ultra-high purity helium (Praxair) was used as the carrier gas at a flow rate of 1.10 ml/minute. Optima HPLC Grade methanol (Thermo Fisher Scientific) was used to wash the injection syringe between each sample.

Each sample underwent a heating ramp in the Trace 1310 that started at 70 °C which was maintained for 2 minutes and then ramped up at a rate of 25 °C/min until reaching 350 °C which was maintained for 5 minutes. The MS transfer line was kept at 280 °C and the ion source was maintained at 230 °C. Electron ionization at 70 eV and a scan time of 0.25 seconds over the range of 50.0-500.0 amu was sufficient for analysis. Mono-isotopic ions for each iteration of 13C labeled palmitate were extracted by through the Xcalibur version 4.4 through the ICIS peak detection algorithm, which utilized minimal smoothing and a maximum baseline of 12 scans for integration. Palmitate retention time was confirmed by standards. Total ion current peaks of different metabolites were normalized to the internal standard 3 deuterium labeled heptadecanoic acid [23].

#### RNA immunoprecipitation

MDA-MB-231 cells were treated with or without IGF-1 with either control siRNA or SRPK2 siRNA as described in the transfection methods. Cells were detached by trypsin and washed twice with 1XPBS. Cell pellets were lysed with RIPA buffer supplemented with a protease inhibitor cocktail and RNAse inhibitor (Invitrogen #10777019) 160 units/mL. Cell lysates were sonicated 3 × 20 seconds on ice and incubated for 30 minutes at 4 °C and pre-cleared with 25 ul of protein A magnetic beads (Invitrogen, #10-003D). Magnetic beads were pre-bound to the antibodies by incubating 5 μg of anti-SRSF-1 (Invitrogen, #PA5-30220) or Rabbit IgG (Cell Signaling Technologies, #2729) with 50 ul of beads for 30 minutes at room temperature. Input samples were collected, and pre-bound beads were incubated with pre-cleared supernatant for 3 hours at 4C. Beads were washed 6 times in RIPA buffer and transferred to 150 ul of RIPA buffer containing of 1% SDS and 1 mg/mL of proteinase K. Beads were eluted by incubating at 50 ° C for 30 minutes. Eluted beads were separated by a magnetic and RNA was extracted using a Qiagen RNAeasy mini kit (Qiagen, #74134) according to manufactures instructions. 500 ng of Immunoprecipitated RNA as well as corresponding input RNA was subjected to cDNA synthesis using the same protocol listed in RT-qPCR section. Exon 4 specific primers were used to amplify cDNA using SYBR Green master mix. Resulting Ct values were analyzed to be expressed as % of transcript bound to the antibody by using the % input method (100 X 2^[Ct (input) - Ct (IP)].

### Statistical analysis

Data obtained from western blot, RT-qPCR, and GC-MS were analyzed using students t-test and done in biological triplicate ±SD or S.E.M. Quantification from immunofluorescence data was analyzed by two-way ANOVA followed by Tukey’s HSD post hoc analysis. More details regarding *p*-values and analysis can be found in the figure legends.

## Results

### IGF-1 mediates FASN expression in breast cancer through mTORC1

**Fig. 1:**
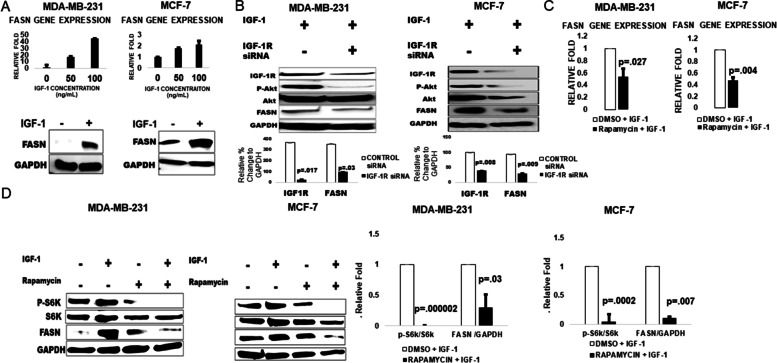
(**A-D)**. IGF-1 mediates FASN expression in breast cancer through mTORC1. **A** MDA-MB-231 and MCF-7 cells were serum starved 16 hours followed by either 0, 50, or 100 ng/mL of IGF-1 exposure for 6 hours in full growth media prior to RT-qPCR analysis. Data are represented as a mean of 3 independent trials ± SD. Values are expressed as a relative fold change to no IGF-1 exposure and were standardized to actin. Western blot analysis below the graphs shows an increase in FASN protein expression following IGF-1 exposure (100 ng/mL) for 24 hours. GAPDH was used as a loading control. Band quantification of the 3 trials (*n =* 3) is expressed as a fold change to no IGF-1 treatment. GAPDH was used to standardize the protein band intensity. All blots in Fig. 1 were cropped to simplify data interpretation. Full blots can be found in Fig. S1. **B** MDA-MB-231 and MCF-7 cells were untreated or transfected with either a control siRNA or IGF-1R siRNA for 48 hours. Cells were then exposed with or without IGF-1 (100 ng/mL) for 24 hours in full growth media prior to western blot analysis. Band quantification is represented as a graph below expressing the percent change of IGF-1R and FASN relative to the GAPDH loading control. Bars are represented as a mean of three band intensities from 3 independent trials (*n =* 3) ± SD. **C** MDA-MB-231 and MCF-7 cells were serum starved 16 hours with vehicle or rapamycin (100 nM) followed by IGF-1 exposure (100 ng/mL) for 6 hours in full growth media prior to RT-qPCR analysis. Graphs are expressed as a fold change to vehicle and represent an average of three independent trials ± SD. All values were standardized to actin. **D** MDA-MB-231 and MCF-7 cells were serum starved with vehicle or rapamycin (100 nM) and exposed to IGF-1 (100 ng/mL) for 24 hours before western blot analysis. Bar graphs to the right represent an average band quantification from three trials (*n =* 3) ± SD. Bars represent a fold change to DMSO treatment. p-S6K band intensity was standardized to total S6k and FASN standardized to GAPDH. All statistical analyses in A-D were performed by students T test

Regulation of lipogenic metabolism through FASN has been studied extensively and is often mediated downstream of receptor tyrosine kinases (RTK) such as fibroblast growth factor receptor (FGFR), epidermal growth factor receptor (EGFR), insulin receptor (IR), and the oncogenic human epidermal growth factor receptor 2 (HER2) [24–28]. Thus, we sought to evaluate the effect of the IGF-1R signaling axis in FASN regulation. IGF-1 exposure contributed to increases in FASN mRNA and protein expression in both MCF-7 and MDA-MB-231 breast cancer cells (Fig. 1A). To further elucidate the specific role of IGF-1R, RNAi mediated silencing of IGF-1R was conducted in response to IGF-1 exposure. IGF-1R knockdown significantly decreased (*p* = .03, MDA-MB-231; *p* = .009, MCF-7) FASN protein expression in both cell lines (Fig. 1B). To further demonstrate IGF-1R induced FASN regulation, we used the mTORC1 inhibitor, rapamycin, to attenuate mTORC1 activity in response IGF-1 exposure. In both cells lines, pre-treatment with rapamycin resulted in significant decreases (*p* = .027, MDA-MB-231; *p* = .004, MCF-7) in IGF-1 induced FASN gene expression compared to vehicle treated cells (Fig. 1C). Additionally, rapamycin significantly decreased (*p* = .007, MDA-MB-231; *p* = .03, MCF-7) FASN protein expression in response to IGF-1 exposure (Fig. 1D). This was accompanied by a significant decrease in p-S6K protein expression (*p <* .001, MDA-MB-231, *p <* .0001, MCF-7) a direct downstream substrate of mTORC1 signaling (Fig. 1D). These results suggest that mTORC1 is involved in IGF-1 induced FASN expression in breast cancer.

### IGF-1-mTORC1 axis contributes to SRPK2 nuclear localization and phosphorylation

**Fig. 2:**
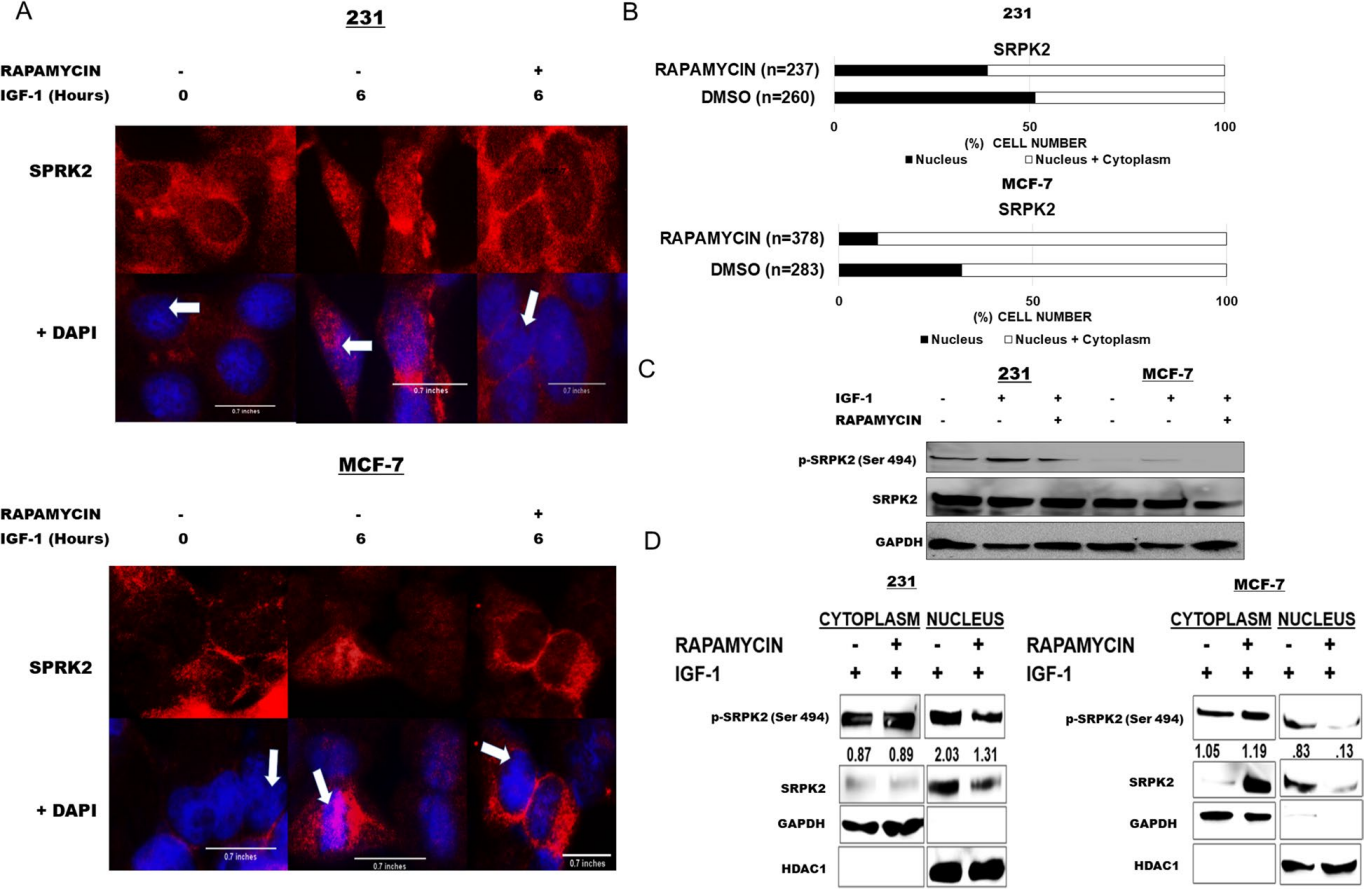
(**A-D**). IGF-1 contributes to SRPK2 nuclear localization and phosphorylation through mTORC1. **A** MCF-7 and 231 cells were serum starved 16 hours in the presence of DMSO or rapamycin (100 nM) followed by exposure to full growth media with or without IGF-1 (100 ng/mL) for various time points. Cells were fixed, permeabilized, and stained for SRPK2 (1:100) followed by anti-mouse Cy3 (Red) (1:1200) antibody. Cells were mounted using mounting medium containing DAPI nuclear stain (Blue). White arrows indicate where the image was zoomed in. Image channels were merged using Image J NIH software and quantified. **B** Quantification of nuclear or nuclear and cytoplasmic SRPK2 was performed using FIJI (Image J) software by analyzing the merged channel images for SRPK2 and DAPI. A threshold was set for the colocalization of DAPI and SRPK2 and cells that were over the threshold were counted. Nuclear positive SRPK2 cells were expressed as a percentage of the total amount of cells present in the image. Experiments were repeated three times and at least 200 cells were counted for each condition. **C** MCF-7 and MDA-MB-231 cells were serum starved overnight (16 hours) with DMSO or rapamycin (100 nM) followed by either full growth media alone or with 100 ng/mL of IGF-1 for 6 hours. Cells were harvested and analyzed by western blot for p-SRPK2, SRPK2, and GAPDH. All blots for fig. 2 were cropped to provide a concise representation of the data. Full blots can be found in Fig. S1. **D** MCF-7 and MDA-MB-231 were serum starved 16 hours with DMSO or rapamycin (100 nM) followed by 6 hours of full media exposure with 100 ng/mL of IGF-1. Cells were harvested and fractionated into both cytosolic and nuclear fractions. Fractions were analyzed by western blot. GAPDH served as the loading control for cytosolic fractions and HDAC1 served as the nuclear fraction loading control. The blot is a representation of 1 trial. Numbers under p-SRPK2 represent an average band intensity from three independent trials (*n =* 3). Band intensity was standardized to respected loading controls, GAPDH (cytoplasm) or HDAC1 (nucleus)

Recent studies have demonstrated the downstream substrate of mTORC1, ribosomal S6 kinase (S6K) in phosphorylating SRPK2 [8, 9]. Phosphorylation of SRPKs can alter their conformation and potentially affect nuclear localization through their nuclear localization signals (NLS) [8, 9]. Thus, we aimed to determine if the IGF-1-mTORC1 axis affects the subcellular localization of SRPK2 in breast cancer cells. To determine the role the IGF-1 mTORC1 pathway and SRPK2 dynamics, breast cancer cells were serum starved overnight in the presence of either mTORC1 inhibitor, rapamycin, or vehicle and exposed to IGF-1 for various time points. IGF-1 induced nuclear localization of SRPK2 at 6 and 12 hours of exposure in both MCF-7 and MDA-MB-231 cell lines (Fig. S2). This was not apparent at hours 2 and 18 of IGF-1 exposure (Fig. S2). At a six-hour time point, there was a substantial increase in nuclear SRPK2 upon IGF-1 exposure in both breast cancer cell lines (Fig. 2A-B), which was abrogated upon rapamycin treatment. Recent work has illustrated the potential of mTORC1 signaling to promote the phosphorylation of SRPK2 and its contribution to its nuclear localization [8, 9]. Thus, we aimed to explore if IGF-1 contributed to phosphorylation of SRPK2. Upon IGF-1 exposure for 6 hours, there was an increase in phosphorylated SRPK2 in both cell lines and this increase in phosphorylation was attenuated by rapamycin treatment (Fig. 2C). We further investigated the subcellular localization of phosphorylated SRPK2 upon IGF- 1 exposure by performing cell fractionation in which breast cancer cells were treated with a vehicle or rapamycin and exposed to IGF-1. Rapamycin reduced the amount of nuclear phosphorylated SRPK2 and increased cytosolic, compared to control in response to IGF-1 exposure (Fig. 2D). Thus, our current findings demonstrate that IGF-1 induces nuclear localization and phosphorylation of SRPK2 through mTORC1.

### SRPK2 contributes to IGF-1-induced FASN expression and de novo fatty acid synthesis

**Fig. 3:**
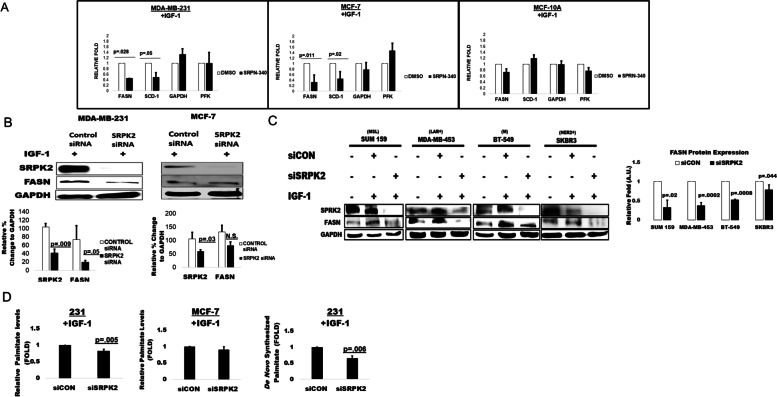
(**A-E**). IGF1-SRPK2 mediates FASN gene expression and de novo lipid synthesis. **A** MCF-7, MDA-MB-231, or MCF-10A cells were serum starved 16 hours followed by vehicle (DMSO) or SRPK2 inhibitor (SRPN-340) (3 μM) pretreatment for 1 hour followed by IGF-1 (100 ng/mL) exposure for 6 hours in full growth media. Graphs represent a relative fold change to vehicle control treatment. All values were standardized to actin and are represented as a mean of three independent trials (*n =* 3) ± SD. Students T test were used to calculate significance. “N.S.” = *p*-value ≥ .05 **B** MCF-7 and MDA-MB-231 cells were transfected with either control siRNA or SRPK2 siRNA prior to IGF-1 exposure (100 ng/mL) for 24 hours. SRPK2, FASN, and GAPDH protein expression were detected by western blot. GAPDH served as the loading control. Graphs to the right of the blots are representative of band quantifications from three independent trials (*n =* 3) ± SD. Students T test were performed as statistical analysis for determining significance. Blots have been cropped for data interpretation and full blots can be found in Fig. S1. **C** SUM-159, MDA-MB-453, BT-549, and SKBR3 cells were transfected with control SRPK2 siRNA with or without IGF-1 exposure. FASN, SRPK2, and GAPDH protein expression were detected by western blot. Graphs below represent an average of three independent trials (*n =* 3) ± SD and expressed as a fold change to control siRNA. Students T test were performed as statistical analysis. Blots have been cropped for data interpretation and full blots can be found in Fig. S1. **D** MDA-MB-231 and MCF-7 cells were transfected with control or SRPK2 siRNAs followed by exposure to media containing charcoal stripped FBS, ^13^c glucose (4.5 g/L), and IGF-1 (100 ng/mL) for 48 hours prior to GC-MS analysis. Graphs represent relative fold change of total intracellular palmitate. Graphs represent an average of 3 independent trials (*n =* 3) ± S.E.M. Unpaired two-way students t-test were performed for statistical analysis

SRPK2 has been demonstrated to regulate lipogenesis through post-transcriptional RNA processing [8, 9]. Thus, we proposed that IGF-1 induced FASN regulation was through the mTORC1-SRPK2 axis. To investigate an IGF-1-SRPK2 mediated FASN regulation, breast cancer cells were treated with vehicle or SRPN-340, a small molecule inhibitor of SRPK2. Interestingly, the inhibition of SRPK2 decreased total mRNA expression of both FASN and sterol-CoA desaturase-1 (SCD-1), another lipogenic enzyme in the de novo fatty acid synthesis pathway but had no significant impact on gene expression of glycolytic enzymes, glyceraldehyde 3-phosphate dehydrogenase (GAPDH) and phosphofructokinase platelet (PFK) (Fig. 3A). Additionally, SRPK2 inhibition had no significant impact on any gene expression in the non-transformed mammary epithelial cell line, MCF-10A, suggesting this a cancer cell specific pathway (Fig. 3A). We further investigated if SRPK2 also contributed to IGF-1 induced FASN protein expression by knocking down SRPK2 by RNAi. Surprisingly, we observed a specific decrease in FASN protein expression upon SRPK2 knockdown only in the MDA-MB-231 breast cancer cells and not int the MCF-7 cells (Fig. 3B). Since MDA-MB-231 cells are triple negative breast cancer (TNBC) cells, we next asked if the IGF-1-SRPK2 axis modulated FASN protein expression in other breast cancer cell lines. To this end, MDA-MB-453 (TNBC, luminal androgen receptor +), SUM-159 (TNBC, mesenchymal stem-like), BT-549 (TNBC, mesenchymal) and SKBR3 (HER2+) cells were transfected with control or SRPK2 specific siRNA followed with IGF-1 exposure. In all of the cell lines, we observed significant decreases in FASN protein expression upon SRPK2 knockdown, suggesting that this observed response was not specific to a single cell line.

Given its role in FASN mRNA and protein expression, we next explored the role of SRPK2 in de novo palmitate synthesis in cancer cells in response to IGF-1 exposure. To investigate the role of SRPK2 in IGF-1 indued palmitate synthesis, we knocked down SRPK2 by RNAi and incorporated [U-^13^ C] glucose into lipid stripped media to measure de novo synthesis. SRPK2 knockdown resulted in significant decreases in total palmitate upon IGF-1 exposure in MDA-MB-231 cells only (Fig. 3C) [14]. Additionally, we observed a significant reduction of full labeled palmitate from [U-^13^ C] glucose (C16:0) in MDA-MB-231 cell lines and not in the MCF-7 (Fig. 3D). These results elucidate the role of a potential IGF-1-SRPK2 FASN axis in breast cancer and that the role of this pathway in palmitate synthesis may be subtype specific.

### The IGF-1-mTORC1-SRPK2 axis contributes to FASN mRNA stability

**Fig. 4:**
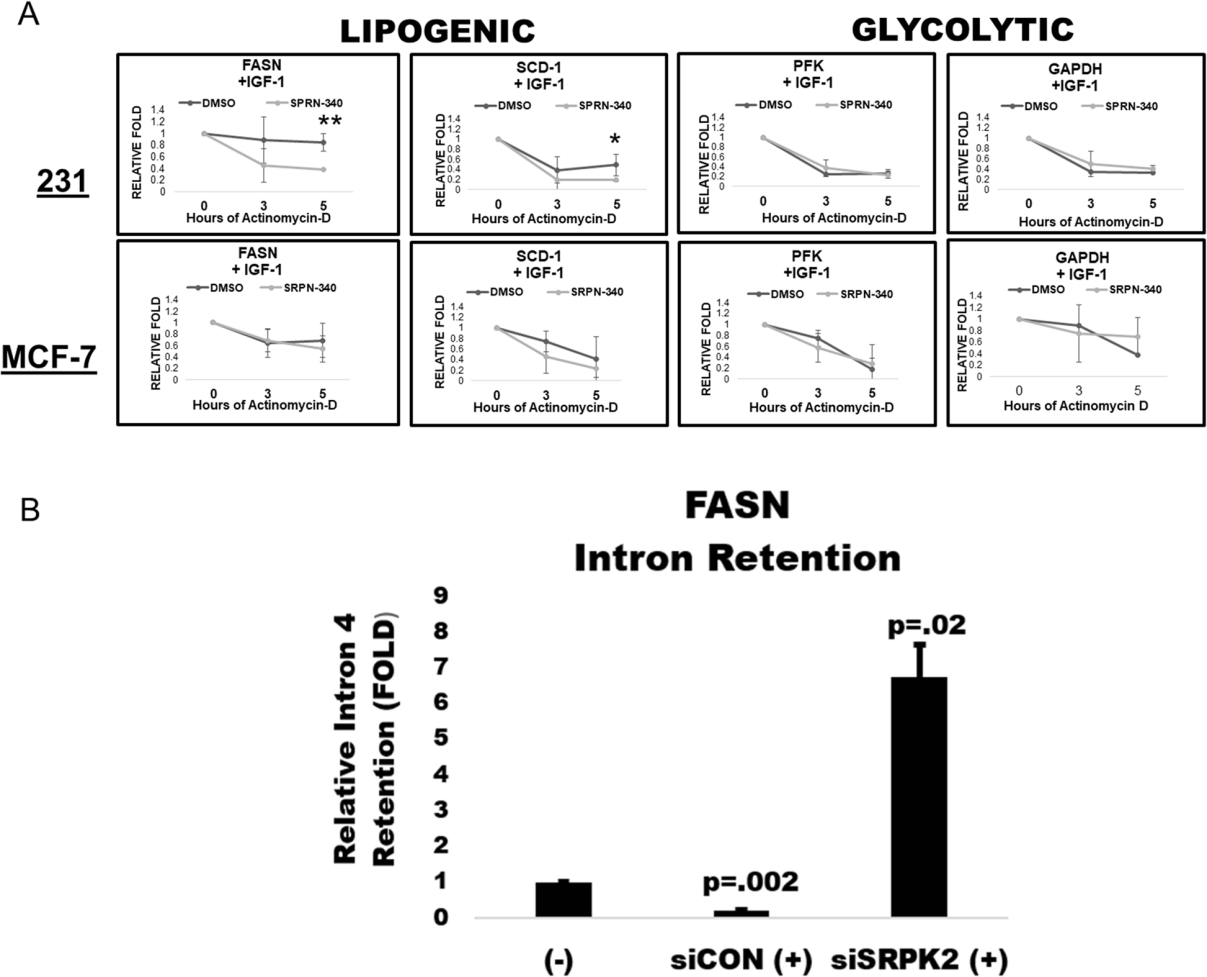
(**A-B**). The IGF-1-SRPK2 AXIS PROMOTE FASN mRNA stability. **A** MDA-MB-231 and MCF-7 cells were serum starved overnight with vehicle or SRPK2 inhibitor (3 μM) followed by exposure to IGF-1 (100 ng/mL) and with Actinomycin-D (5 μg/ mL) for 0, 3, 5 hours in full growth media, prior to RT-qPCR analysis. All values were standardized to actin. Graphs represent a relative fold change to baseline (0 hours of actinomycin-D) are expressed as means of three independent trials (*n =* 3) ± SD. Students T test were performed to determine significance. “*” = *p* ≤ .05, “**” = *p* ≤ .01, “N.S” = *p* ≥ .05. **B** MDA-MB-231 cells were transfected with control or SRPK2 siRNA for 48 hours and serum starved for 24 hours with or without IGF-1 (100 ng/mL) exposure. RNA was subjected to RT-qPCR with FASN intron or exon specific primers. Significance testing were done using the mean folds of three independent trials (*n =* 3) ± S.E.M. Student’s T test was performed for statistical analysis

SRPK2 induces lipogenic gene expression through pre-mRNA splicing, primarily mediated through the phosphorylation of RNA binding proteins, serine/arginine rich splicing factors (SRSFs), and can result in mRNA stabilization [8, 9]. Thus, we sought to investigate if IGF-1 could mediate FASN mRNA stability through our observed IGF-1-mTORC1- SRPK2 pathway. To ask if IGF-1 induces mRNA stability through SRPK2, breast cancer cells were exposed to IGF-1 with a vehicle or SRPK2 inhibitor, SRPN-340, prior to treatment with actinomycin-D, a transcriptional inhibitor, for 0, 3, and 5 hours [8]. In response to IGF-1 exposure, we observed a significant decrease in mRNA stability in both FASN and SCD-1 upon SRPK2 inhibition, which was specific only to the MDA-MB-231 cells (Fig. 4A). Additionally, there was no significant effect on the mRNA stability of glycolytic PFK and GAPDH in the MDA-MB-231 cells (Fig. 4A). Intron retention is alternative splicing event, which can result in mRNA decay [9]. Thus, we investigated if this observed decrease in mRNA stability was due to an increase in intron retention. To this end, we transfected MDA-MB-231 cells with control or SRPK2 siRNA and performed an intron retention analysis using FASN intron specific primers and RT-qPCR. Interestingly, we found that IGF-1 significantly reduced intron retention, which was rescued by SRPK2 knockdown in the MDA-MB-231 cells (Fig. 4B). we which was abolished upon SRPK2 knockdown (Fig. 4B). These findings propose that IGF-1 regulates FASN through abrogating IR, which is dependent upon SRPK2.

### The IGF-1mTORC1-SRPK2 axis regulates FASN through SRSF-1

**Fig. 5:**
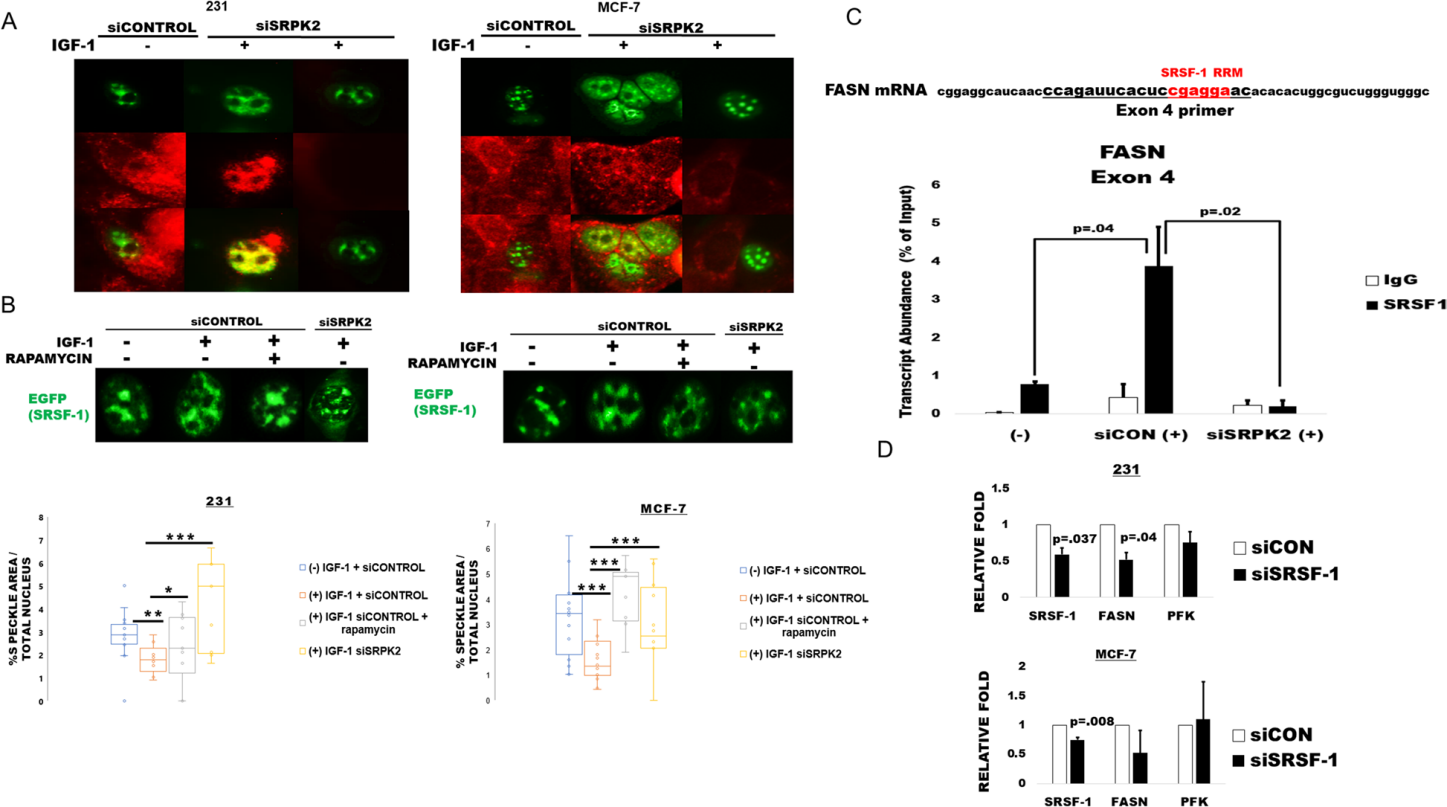
The IGF-1-mTORC1-SRPK2 axis mediates FASN regulation through SRSF-1 **A** MDA-MB-231 and MCF-7 cells were grown in 8-well chamber slides and co-transfected with pSRSF-1-EGFP and either control or SRPK2 siRNA for 24 hours prior to treatment with vehicle or rapamycin. Cells were exposed with or without IGF-1 (100 ng/mL) for 6 hours before being fixed for subsequent fluorescence microscopy analysis. Fixed cells were incubated with anti-SRPK2 or anti-P-SRPK2(Ser474) primary antibodies followed by Cy3 conjugated secondary antibodies (Red). Nuclei were stained with DAPI nuclear stain (Blue) that is present in the mounting media. Images were obtained on a Leica confocal microscope. Trials were repeated at least 2 times. **B** MCF-7 and MDA-MB-231 cells were co-transfected with SRSF-1-eGFP plasmid with either control siRNA or SRPK2 siRNA for 48 hours. Cells were treated with vehicle or (100 nM) rapamycin for 16 hours prior to being exposed with or without IGF-1 for an additional 6 hours. Fixed cells were incubated with either SRPK2 or p-SRPK2 antibody followed by CY3 conjugated secondary and mounted with a DAPI nuclear stain containing medium. Nuclear speckle area was measured using image J and standardized to total nuclear area. Graphs are representative of at least 20 speckles from 3 independent trials. Average nuclear speckle/total nuclear area arbitrary units were used to construct box plots below [15, 16]. Statistical analysis was performed using one-way ANOVA followed by a Tukey’s post hoc analysis. Bars above box plots represent the significance between the respected conditions. Codes for significance between groups are as follows: *p <* .05 = * *p <* .01 = **, *p <* .001 = ***. **C** MDA-MB-231 transfected cells were exposed with (+) or without (−) IGF-1 for 6 hours followed by RNA-IP with SRSF-1 or control (IgG) antibodies. RNA was eluted from the antibodies and expressed as % of antibody bound transcript. Bars represent an average of three independent trials (*n =* 3) ± SD. Student’s T test were performed for statistical analysis. **D** MCF-7 and MDA-MB-231 cells were transfected with control or SRSF-1 siRNA for 48 hours, serum starved for 16 hours in the presence of 100 ng/mL of IGF-1 for an additional 6 hours. RNA was subjected to RT-qPCR. Graphs represent mean fold changes from three independent trials (*n =* 3). FASN, PFK, and SRSF-1 expression was standardized to actin and normalized to the control siRNA group to be expressed as a relative fold change. Students t test were performed for statistical analysis

Given our results demonstrating IGF-1 induced SRPK2 nuclear localization, we proposed that IGF-1 also mediates nuclear localization of SRSF-1, a common SRPK2 substrate and SR protein involved in pre-mRNA processing [8, 10]. SRSF-1 was expressed in breast cancer cells with a p-eGFP-C1 vector (EGFP-SRSF-1), which has been shown previously to be highly homologous with endogenous SRSF-1 localization and function [15, 29]. To our surprise, SRSF-1 was predominantly localized to the nucleus regardless of IGF-1 exposure (Fig. 5A). Pre-mRNA splicing machinery including SR proteins, hnRNPs, and snRNPs are stored in membrane-free structures, termed nuclear speckles [16]. Interestingly, we observed consistent decreases in EGFP-SRSF-1 distribution as well as more diffusion within the nucleus upon IGF-1 exposure (Fig. 5B). Co-transfecting cells with an SRPK2 siRNA, attenuated the IGF-1 induced decrease the observed SRSF-1 speckle-like size (Fig. 5A). Additionally, treatment with rapamycin had similar effects (Fig. 5B). These results suggest that the IGF-1-mTORC1-SRPK2 axis alters SRSF-1 nuclear localization. When released from speckles, SR proteins are able to initiate pre-mRNA splicing of nascent RNA transcripts through their RNA recognition motifs (RRMs) [16]. To determine if IGF-1 was mediating the binding of SRSF-1 to FASN pre-mRNA, we performed RNA-immunoprecipitation. Intriguingly, we observed that IGF-1 exposure significantly increased the binding of SRSF-1 to FASN mRNA, which as abrogated by SRPK2 knockdown (Fig. 5C). Considering SRPK2 knockdown enhanced FASN IR in response to IGF-1 exposure (Fig. 4B), we proposed that SRSF-1 could be mediating FASN regulation downstream of SRPK2. To investigate the role of IGF-1 induced SRSF-1 FASN regulation, we knocked down SRSF-1 by RNAi in breast cancer cells and performed RT-qPCR for FASN mRNA expression. As expected, knocking down SRSF-1 greatly reduced FASN mRNA expression, which little effect on glycolytic expression of PFK (Fig. 5D). However, these effects were specific to MDA-MB-231 cells and not in MCF-7. These results suggest that the IGF-1-SRPK2 axis in MDA-MB-231 cells mediates FASN mRNA regulation is through SRSF-1.

## Discussion

Cancer is characterized by accentuated and aberrant metabolic programming to achieve demands for biomolecule synthesis [3]. Enhanced de novo fatty acid synthesis allows for the greater availability of fatty acids for post-translational lipidation of proteins, energy through mitochondrial beta oxidation, and membrane phospholipids [7, 24]. Moreover, much clinical advancement has been made in targeting FASN and the de novo lipid synthesis pathway in numerous cancers including breast [1, 30, 31]. Thus, understanding regulatory networks in this metabolic pathway is crucial for identifying potential mechanisms of treatment resistance and alternate therapeutic targets. In the present study, we report a potential IGF-1 induced metabolic programming of de novo fatty acid synthesis in triple negative breast cancer cells through FASN.

RTKs including EGFR and FGFR, have been demonstrated in regulating lipogenic metabolism through the stabilization and enhanced processing of sterol-response element binding proteins (SREBP) transcription factors [24]. However, the effect of RTKs on post-transcriptional regulation of lipogenesis has not been explored. Here, we demonstrated the IGF-1/IGF-1R signaling axis to induce FASN protein and mRNA expression in breast cancer cells. Further knockdown of IGF-1R also greatly reduced FASN protein, confirming these findings. RTKs often signal downstream through both PI3K/Akt and Ras/Raf/MAPK pathways that both converge on the mTORC1 signaling axis. Both Akt and ERK have been shown to activate mTORC1 by direct phosphorylation of catalytic subunits or the inhibition of its negative regulatory tuberous sclerosis complex 1 and 2 (TSC ½) [8]. We observed a substantial decrease in both FASN and p-Akt upon, IGF-1R knockdown. Further, we demonstrated mTORC1 inhibition to greatly reduced FASN protein and gene expression upon IGF-1 exposure, suggesting that mTORC1 was the responsible downstream pathway involved in IGF-1 induced FASN regulation. We did observe a slight increase FASN protein between the rapamycin and control treatments without IGF-1 exposure, which we propose was due to a feedback mechanism of rapamycin through mTORC2 by promoting Akt Serine 473 phosphorylation. These slight increases in FASN protein expression were not substantial and rapamycin still significantly decreased FASN protein expression upon IGF-1 exposure. Recent studies have shown S6k, the direct mTORC1 substrate, to regulate the expression of lipogenic genes including FASN both transcriptionally and post-transcriptionally [8]. Though we did not study S6K directly, we did find p-S6K to correlate highly with FASN protein expression.

Previous studies have elucidated the role of mTORC1 and SRPK2 nuclear localization [8]. In coordination with these findings, we demonstrated an IGF-1 induced nuclear localization and phosphorylation of SRPK2. Additionally, this IGF-1 induced phosphorylation and nuclear localization was abrogated by treatment with the mTORC1 inhibitor, rapamycin. Other findings have demonstrated the effect of the serine 494 phosphorylation by generating serine 494 to alanine (S494A) mutants [8, 9]. Consistent with our results, these mutants displayed attenuated nuclear localization [8, 9]. Thus, this serine 494 phosphorylation could explain our finding that rapamycin had an accentuated effect in preventing nuclear p-SRPK as opposed to total SRPK upon IGF-1 exposure. Though we did observe significant decreases in total and de novo palmitate synthesis in the triple negative cell line, MDA-MB-231, we did not observe any significant alterations in palmitate synthesis in the ER+ cell line, MCF-7 upon SRPK2 knockdown. This is consistent with our observations that SRPK2 knockdown does not significantly affect FASN protein expression in MCF-7 cell. Additionally, we did demonstrate a significant decrease in FASN and SCD-1 mRNA expression upon treatment with the SRPK inhibitor, SRPN-340. We propose that the results were from off target effects of SRPN-340, which can also inhibit SRPK1. Further, investigation is warranted to elucidate differences between SRPK1 and 2 in mediating lipogenic programming. Given that we observed this affect in three additional triple-negative breast cancer cell lines, these results demonstrate that this modulation of FASN and palmitate synthesis from this pathway could be subtype specific.

Previous work demonstrated that mutating multiple SRPK2 phosphoserine residues results in an increase in intron retention IR [9]. Additionally, both SRPK2 knockdown and mTORC1 inhibition increased the retainment of introns containing premature termination codons (PTC) in lipogenic transcripts, which can initiate NMD and decrease mRNA stability [8]. Consistent with these findings, we observed a decrease in FASN IR upon IGF-1 treatment. This would be consistent with p-SRPK2 reducing FASN IR, given IGF-1 induces several signaling cascades resulting in mTORC1 activation.

SRPK2 regulates pre-mRNA splicing through the phosphorylation of serine-arginine rich splicing factors, SRSFs [8, 11, 32]. In the present study, we found IGF-1 to induce more diffuse SRSF-1 in the nucleoplasm. Pre-mRNA splicing factors are enriched in nuclear speckles and can be released upon phosphorylation [16]. Recent studies have shown that SRPK1 promotes SRSF-1 release from nuclear speckles to the nucleoplasm, decreasing its interaction with cdc2-like kinase 1 (CLK1) [16]. CLK1 acts to maintain SR proteins within the nuclear speckles, preventing its mobilization to the nucleoplasm and subsequent recruitment of the spliceosome [16]. Thus, our results demonstrating IGF-1 induced SRSF-1 diffusion could potentially be through SRPK2 mediated phosphorylation of mobilization of SRSF-1 from nuclear speckles. Inhibition of mTORC1 and/or knockdown of SRPk2 also contributed greatly to accumulation of speckle-like formations of SRSF-1 upon IGF-1 exposure. Thus, the IGF-1-mTORC1-SRPK2 could promote SRSF-1 release from speckles. Though we did observe an alteration in speckle distribution upon IGF-1 exposure in MCF-7 s, we did not demonstrate any effect of SRSF-1 on FASN mRNA expression. These results suggest that IGF-1 does affect SRPK2-SRSF-1 dynamics in MCF-7 cells, but not their ability to regulate FASN. We also found that SRSF-1 knockdown to contribute to a decrease in FASN expression with no significant effect on glycolytic PFK expression, suggesting SRSF-1 as the mediator of the IGF-1-mTORC1-SRPK2-FASN signaling axis demonstrated in the current study. Thus, to our knowledge, this is the first study demonstrating an IGF-1 induced localization of SRSF-1 in breast cancer and its ability to regulate FASN expression.

Based on these current findings, an IGF-1-mTORC1-SRPK2 axis contributes to lipogenic metabolic regulation through FASN in triple negative breast cancer cells. Both SRPK2 and FASN are overexpressed in numerous cancers, suggesting potential therapeutic potential. Additional investigation should be conducted on the specifics behind SRSF-1 induced FASN regulation. Moreover, the consequences of increased FASN expression in breast cancer should also be considered for future directions.

## Supplementary Information

**Additional file 1.**

**Additional file 2.**

**Additional file 3.**

## Data Availability

Any data not presented will be made available upon request. Linda deGraffenried may be contacted for data availability. Email: degraffenried@austin.utexas.edu

## Abbreviations

FASN: Fatty Acid Synthase Enzyme
RTK: Receptor Tyrosine Kinase
SRPK2: Serine-Arginine Rich Protein Kinase 2
SRSFs: Serine-Arginine Rich Splicing Factors
RT-qPCR: Reverse Transcription-quantitative Polymerase Chain Reaction
IGF-1: Insulin-Like Growth Factor 1
IGF-1R: Insulin-Like Growth Factor 1 Receptor
mTORC1: Mammalian Target Of Rapamycin Complex 1
EGFP: Enhanced Green Fluorescent Protein
HER2: Human Epidermal Growth Factor Receptor
SCD: Sterol-CoA Desaturase
ELOVL: Elongases
HBP: Hexosamine Biosynthetic pathway
snRNP: Small Nuclear ribonuclear proteins
hnRNP: Heterogenous nuclear ribonuclear protein
SR domain: Serine Arginine Rich domain
RRM: RNA Recognition Motif
ESE: Exon Splicing Enhancer
IR: Intron Retention
NMD: Nonsense Mediated Decay
EGF: Epidermal Growth Factor
FGF: Fibroblast Growth Factor
ER: Estrogen Receptor
TNBC: Triple Negative Breast Cancer
FBS: Fetal Bovine Serum
MLMTV: Moloney Murine Leukemia Virus
dNTPs: Deoxynucleotide Triphosphate
cDNA: Complementary Deoxynucleic Acid
dCT: Delta Cycle Threshold
ddCT: Delta Delta Cycle Threshold
DMSO: Dimethyl Sulfoxide
SDS-PAGE: Sodium dodecyl Sulfate- Polyacrylamide Gel Electrophoresis
TBST: Tris Buffered Saline and Tween buffer
GAPDH: Glyceraldehyde 3-phosphate dehydrogenase
S6K: S6 Protein Kinase
HRP-linked IgG: Horse Radish Peroxidase linked Immunoglobulin
DAPI: 4′,6-diamidino-2-phenylindole
HDAC1: Histone Deacetylase 1
AKT: Protein Kinase B
GC-MS: Gas Chromatography- Mass Spectrometry
RIPA: Radio Immunoprecipitation Assay
ANOVA: Analysis of Variance
SD: Standard Deviation
SiRNA: Small Interfering Ribonucleic Acid
PFK: Phosphofructokinase Platelet
RAF: Protooncogene c-Rapidly Accelerated Fibrosarcoma
MAPK: Mitogen Activated Protein Kinase
SER: Serine
TSC: Tuberous Sclerosis Complex
CLK: CDC2-Like Kinase
