# Supplementary material for: An IGF-1R-mTORC1-SRPK2 signaling Axis contributes to FASN regulation in breast cancer
Source: BMC Cancer. 2022 Sep 12;22:976. doi: 10.1186/s12885-022-10062-z (PMC9469522; doi:10.1186/s12885-022-10062-z)

Figure 1(A) MCF-7

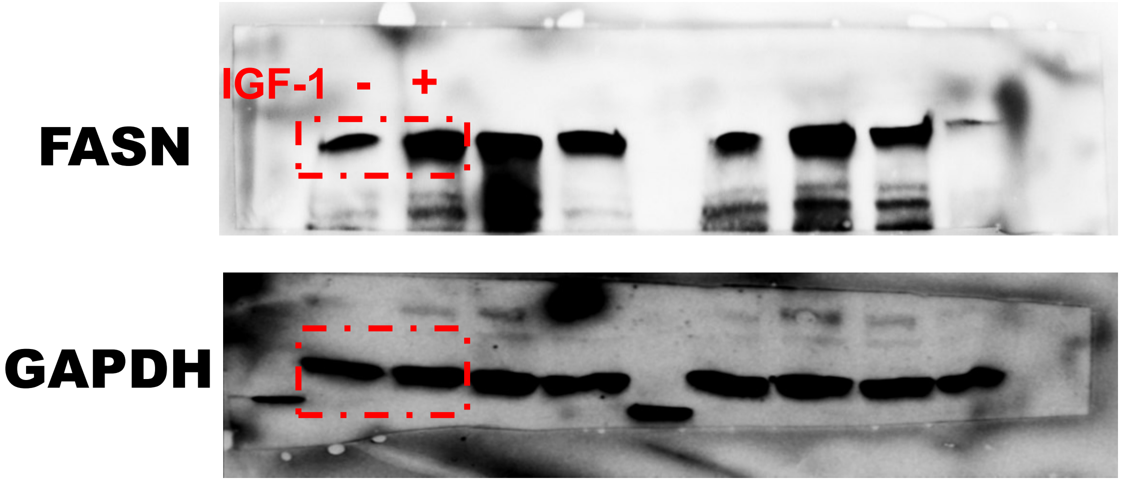

Figure 1(A) MDA-MB-231

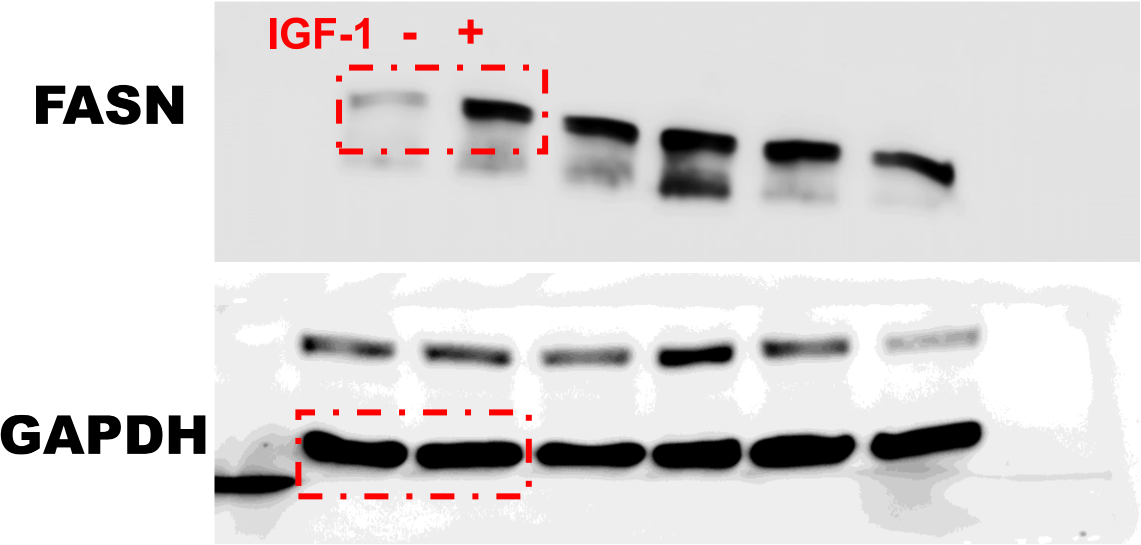

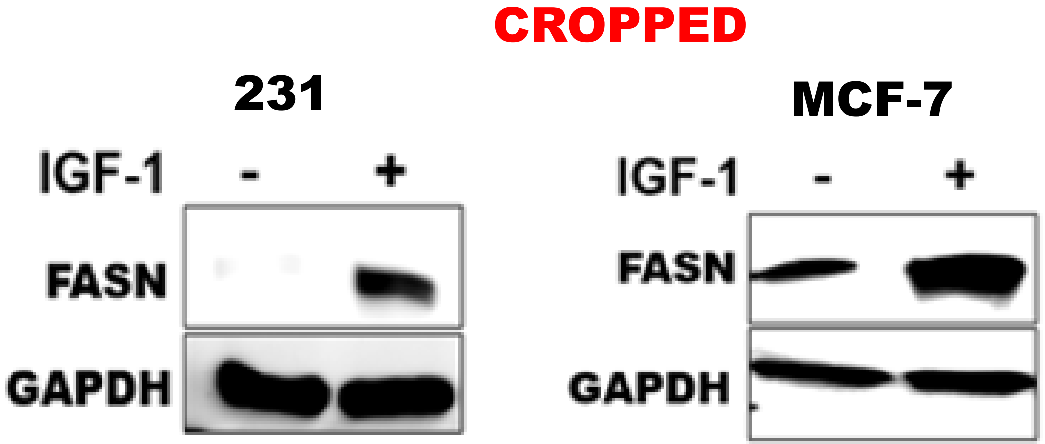

Figure 1(B) MDA-MB-231

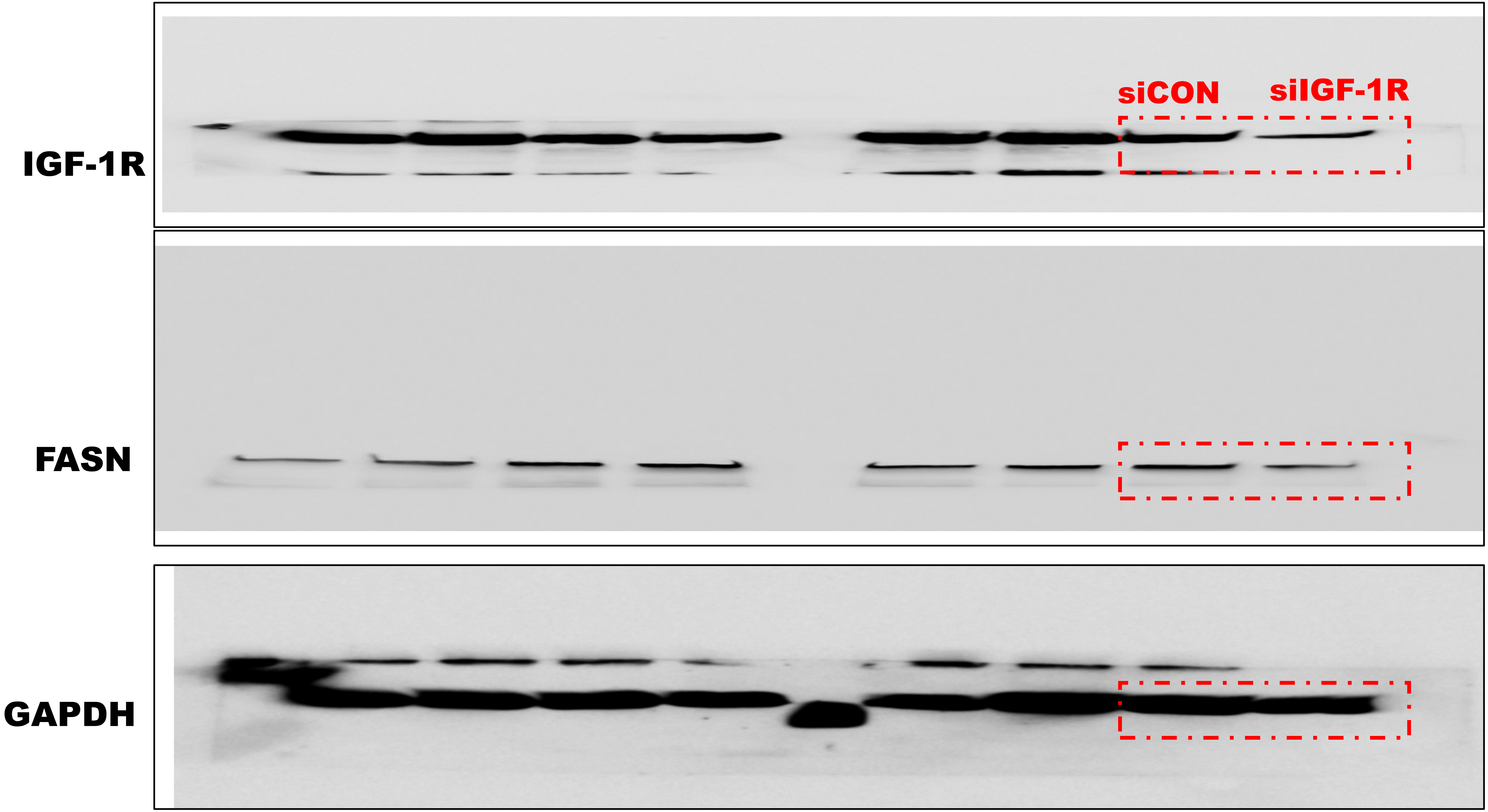

Figure 1(B) MDA-MB-231 (CONTINUED)

LEFT SIDE CUT

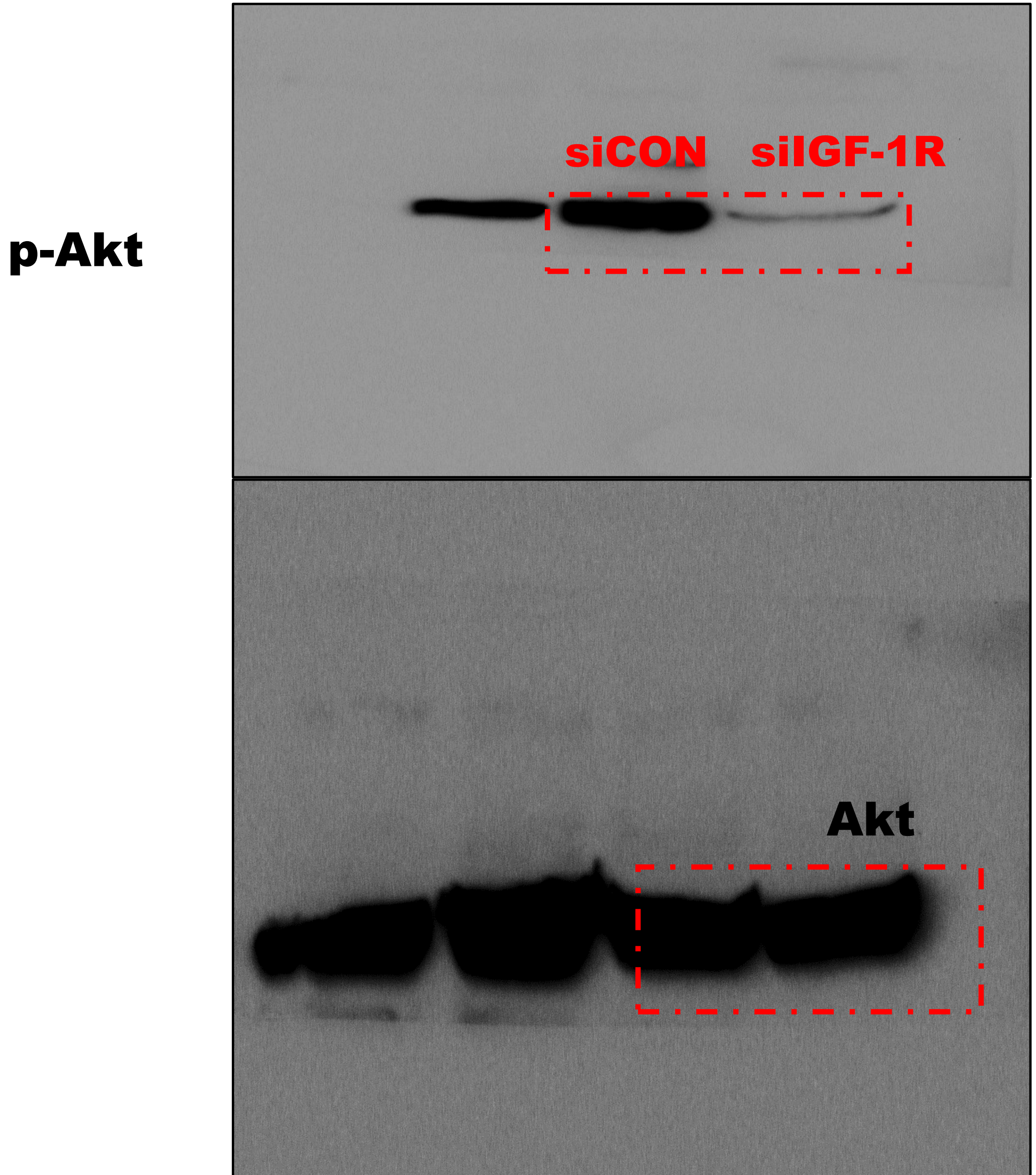

LEFT SIDE CUT

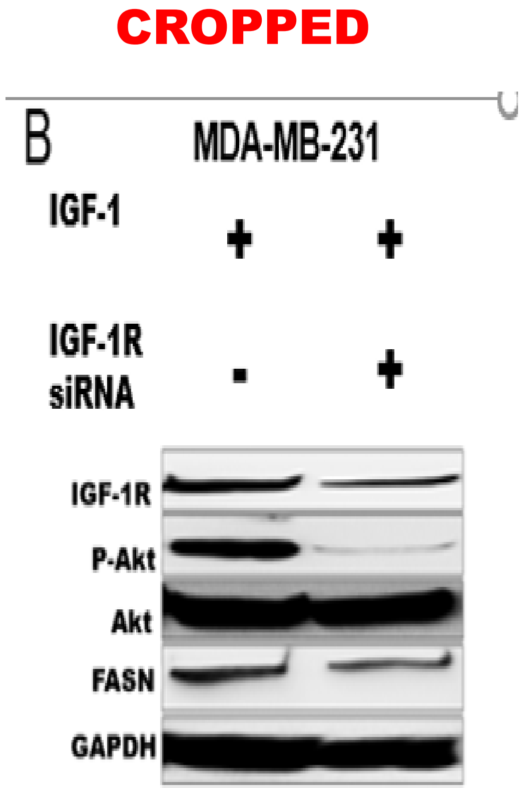

Figure 1(B) MCF-7

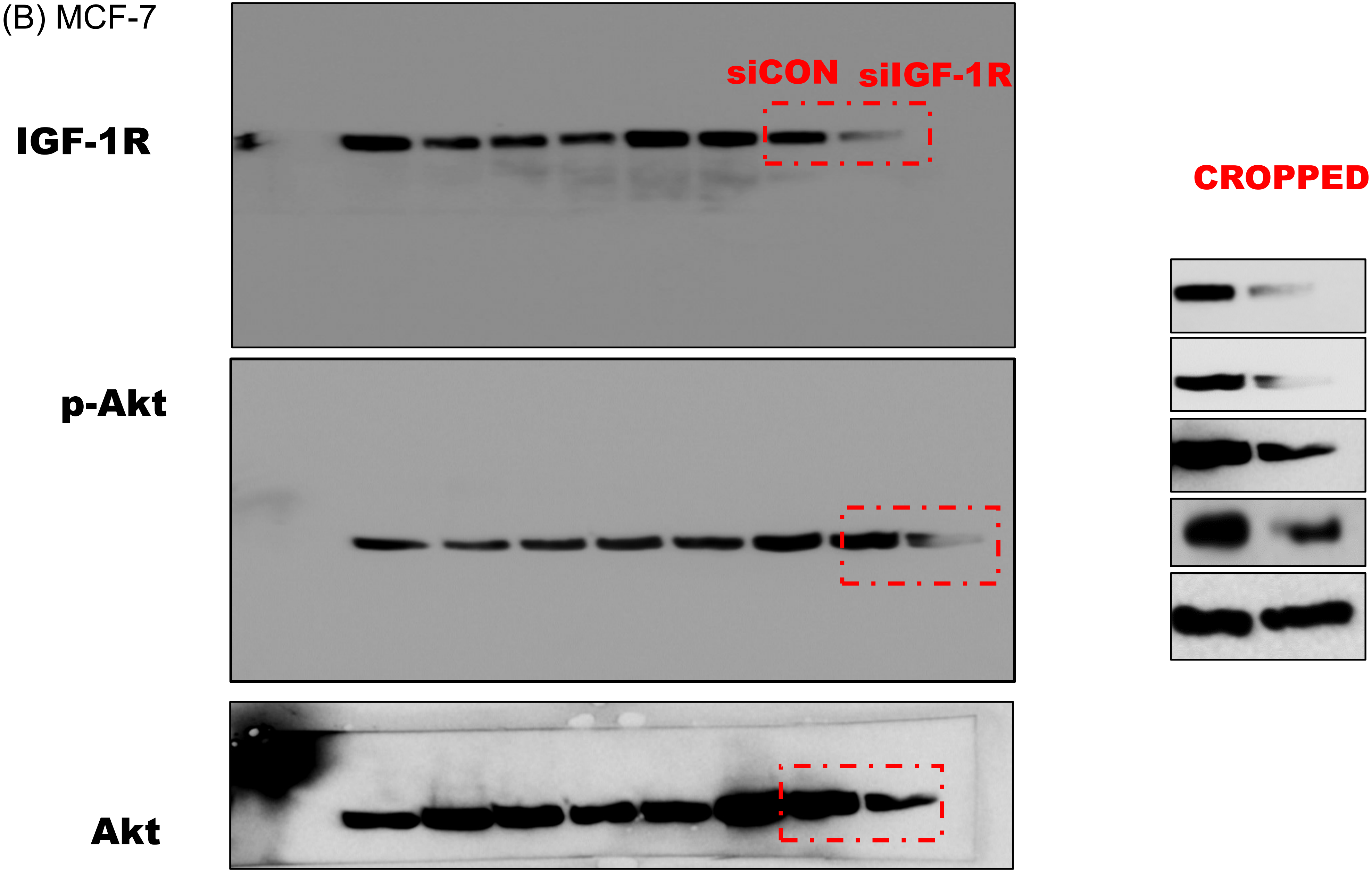

Figure 1(B) MCF-7 (CONTINUED)

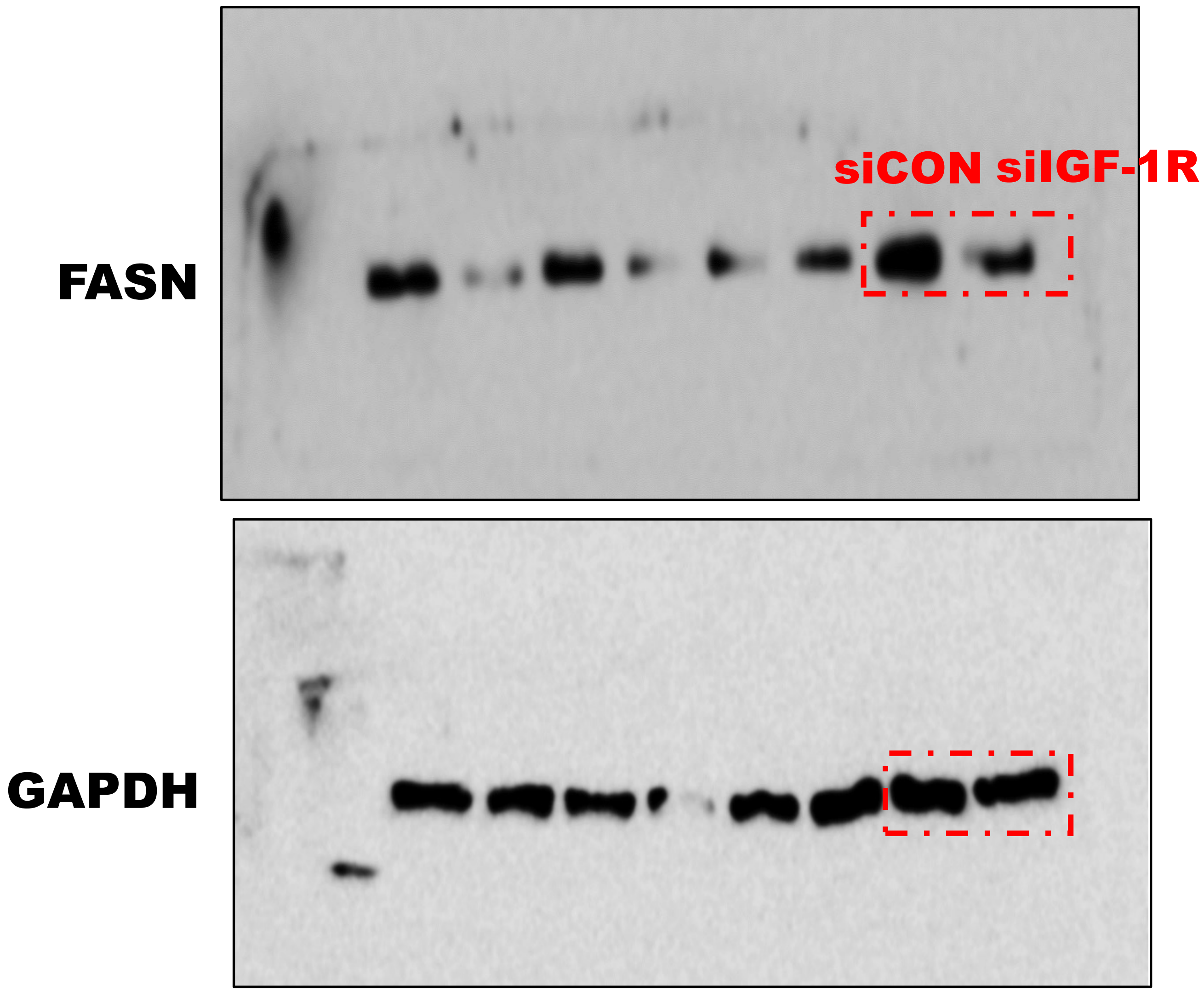

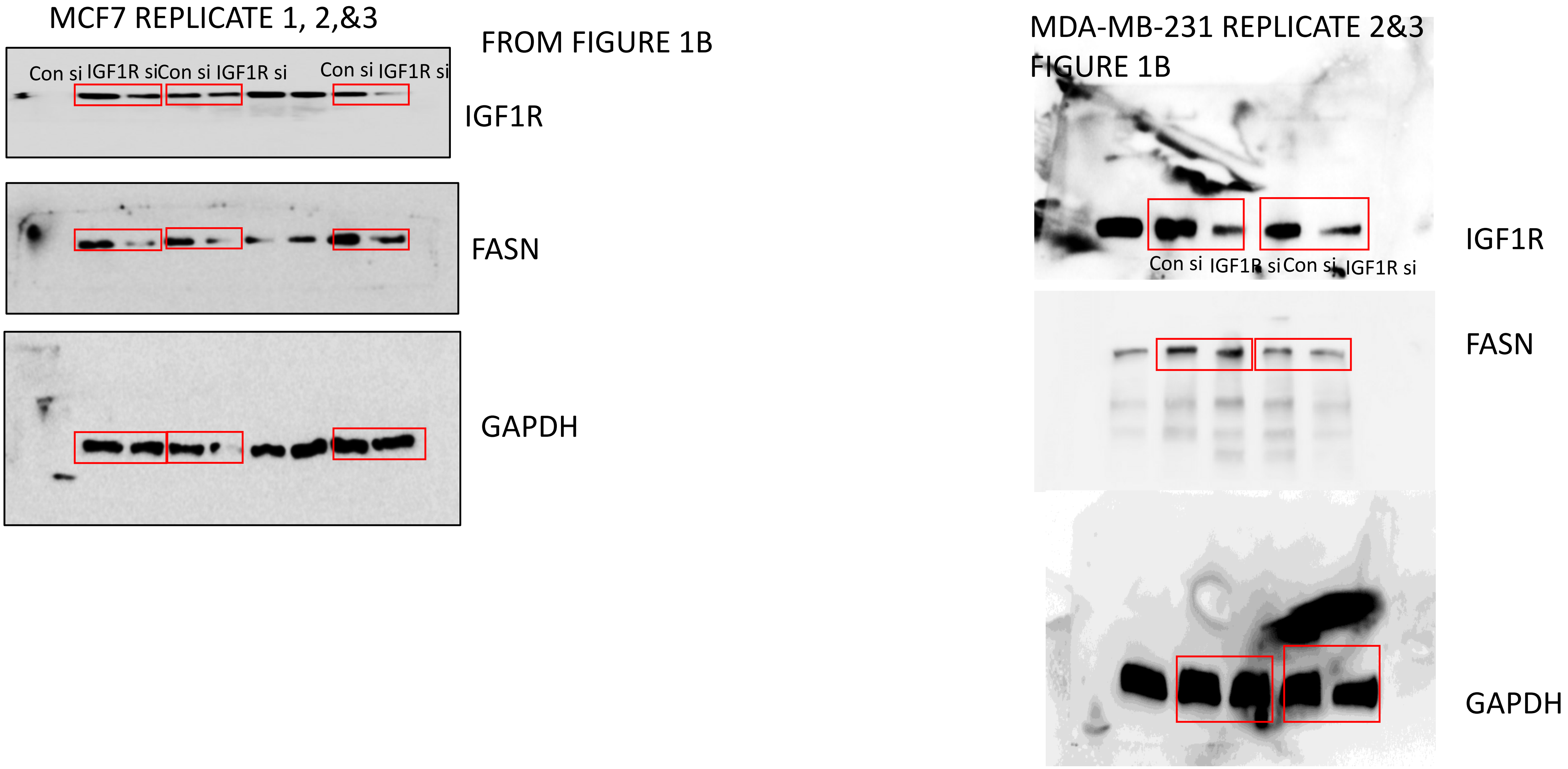
MCF7 REPLICATE 1, 2,&3
FROM FIGURE 1B
Con si IGF1R si Con si IGF1R si Con si IGF1R si
IGF1R
FASN
GAPDH
MDA-MB-231 REPLICATE 2&3
FIGURE 1B
IGF1R
Con si IGF1R si Con si IGF1R si
FASN
GAPDH

Figure 1(D) MCF-7

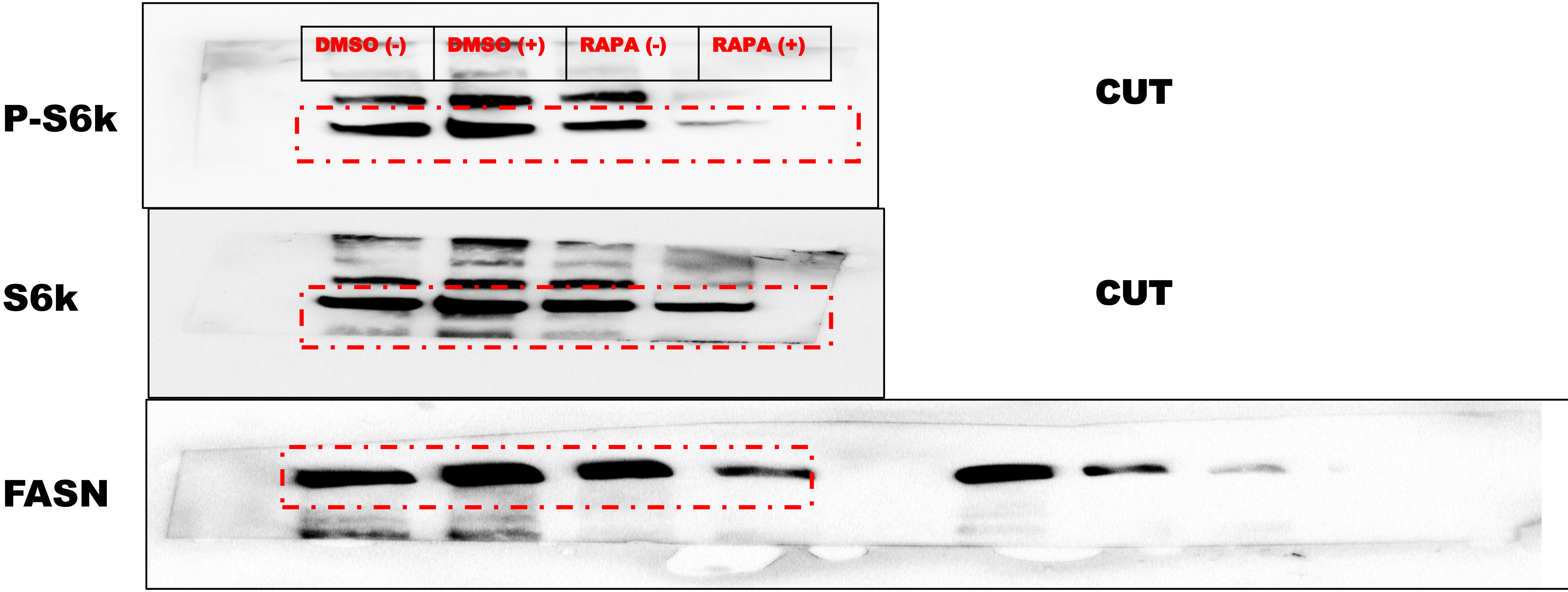

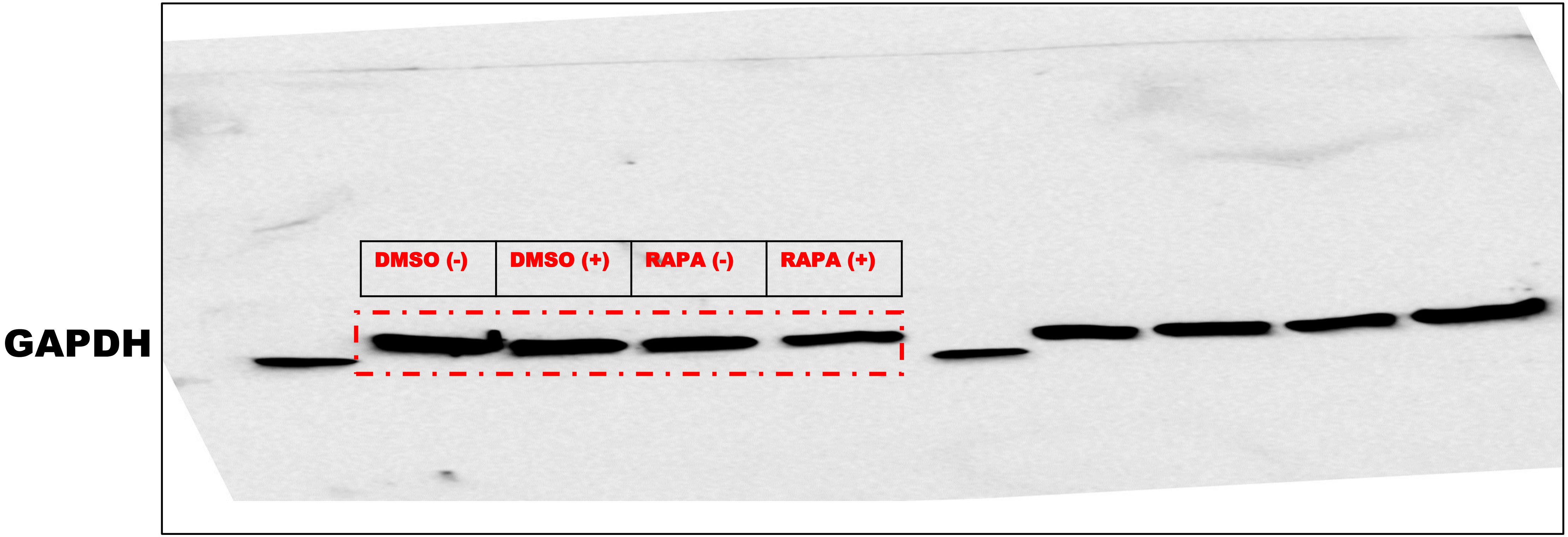

CROPPED

MCF-7

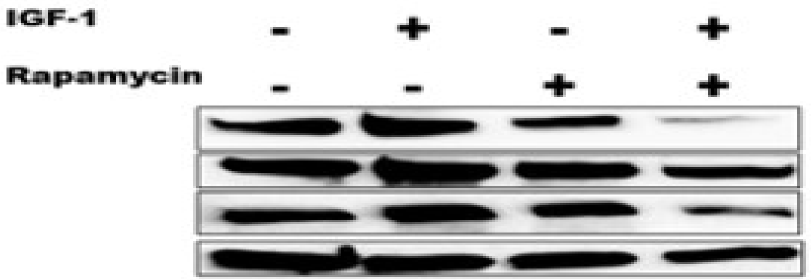

Figure 1(D) MDA-MB-231

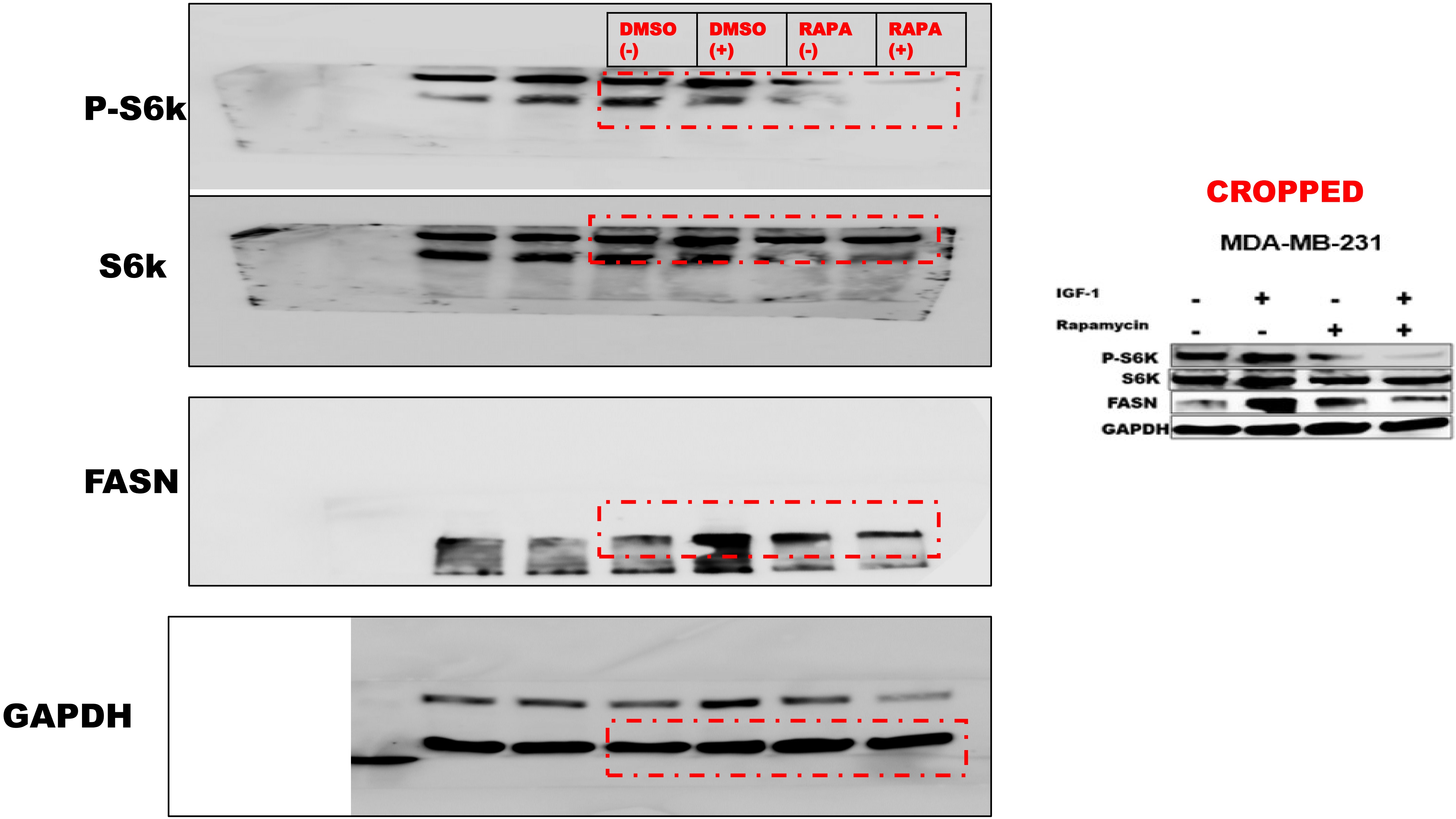

FIGURE 1 D REPLICATE TRIALS

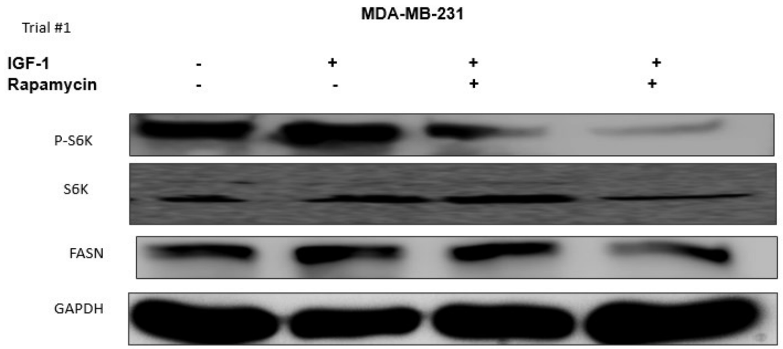

1

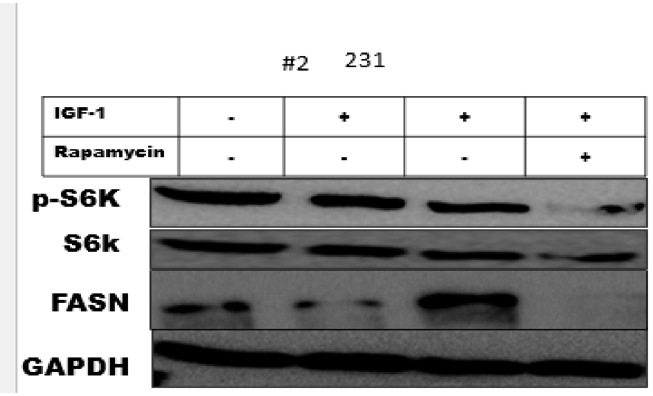

2

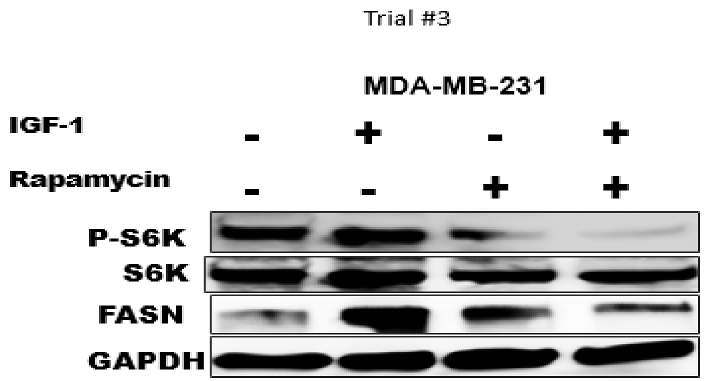

3

1

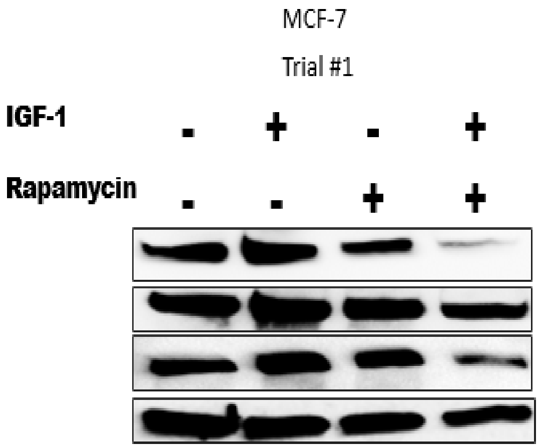

2

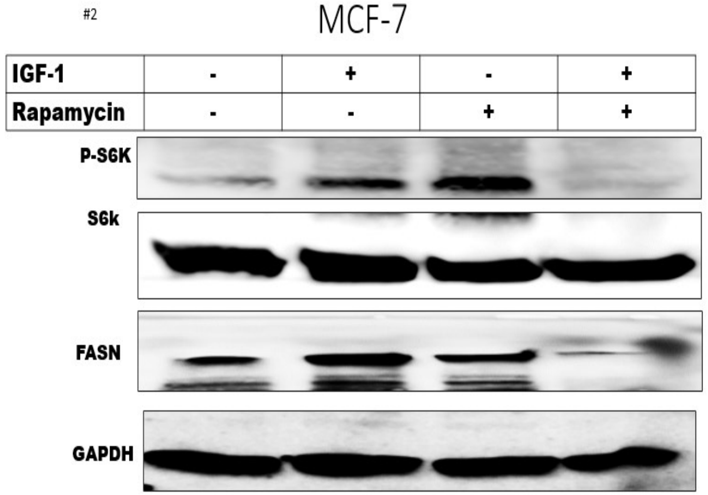

3

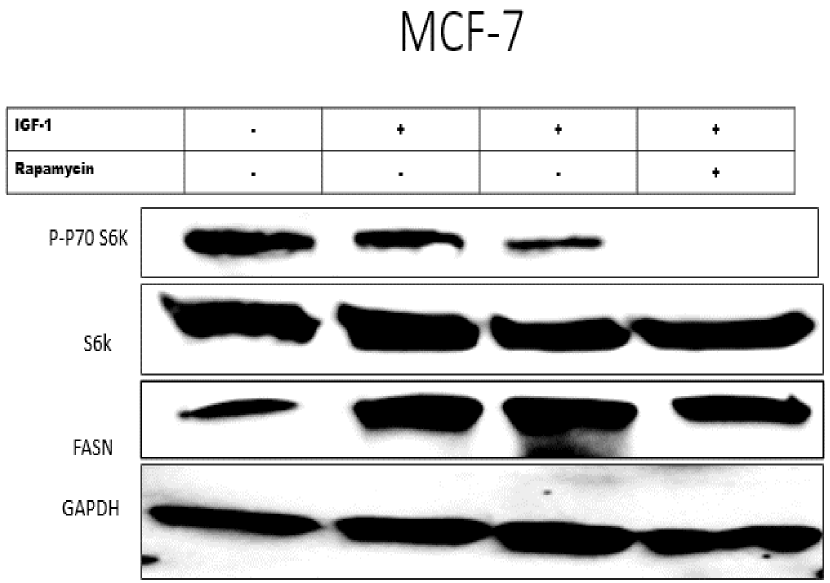

Figure 2(C)

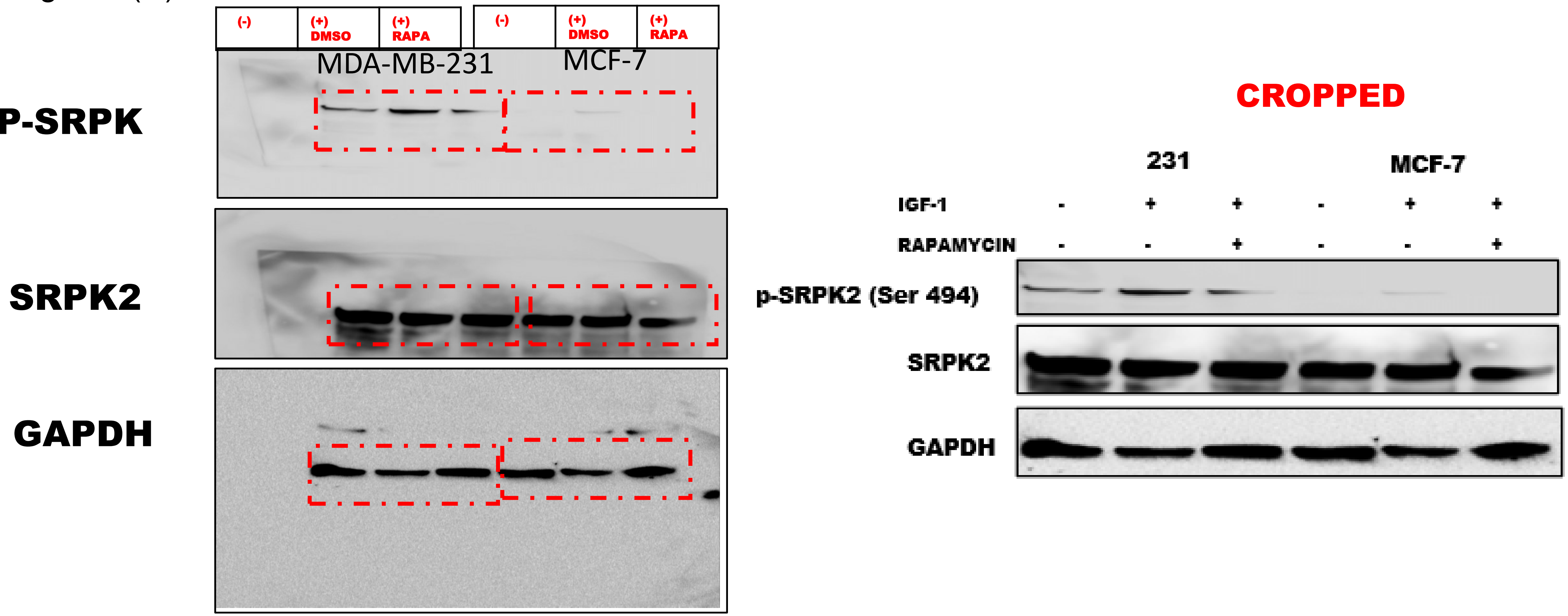
(-)
(+) DMSO
(+) RAPA
(-)
(+) DMSO
(+) RAPA
MDA-MB-231
MCF-7
P-SRPK
SRPK2
GAPDH
CROPPED
231
MCF-7
IGF-1
RAPAMYCIN
p-SRPK2 (Ser 494)
SRPK2
GAPDH

Figure 2(D)

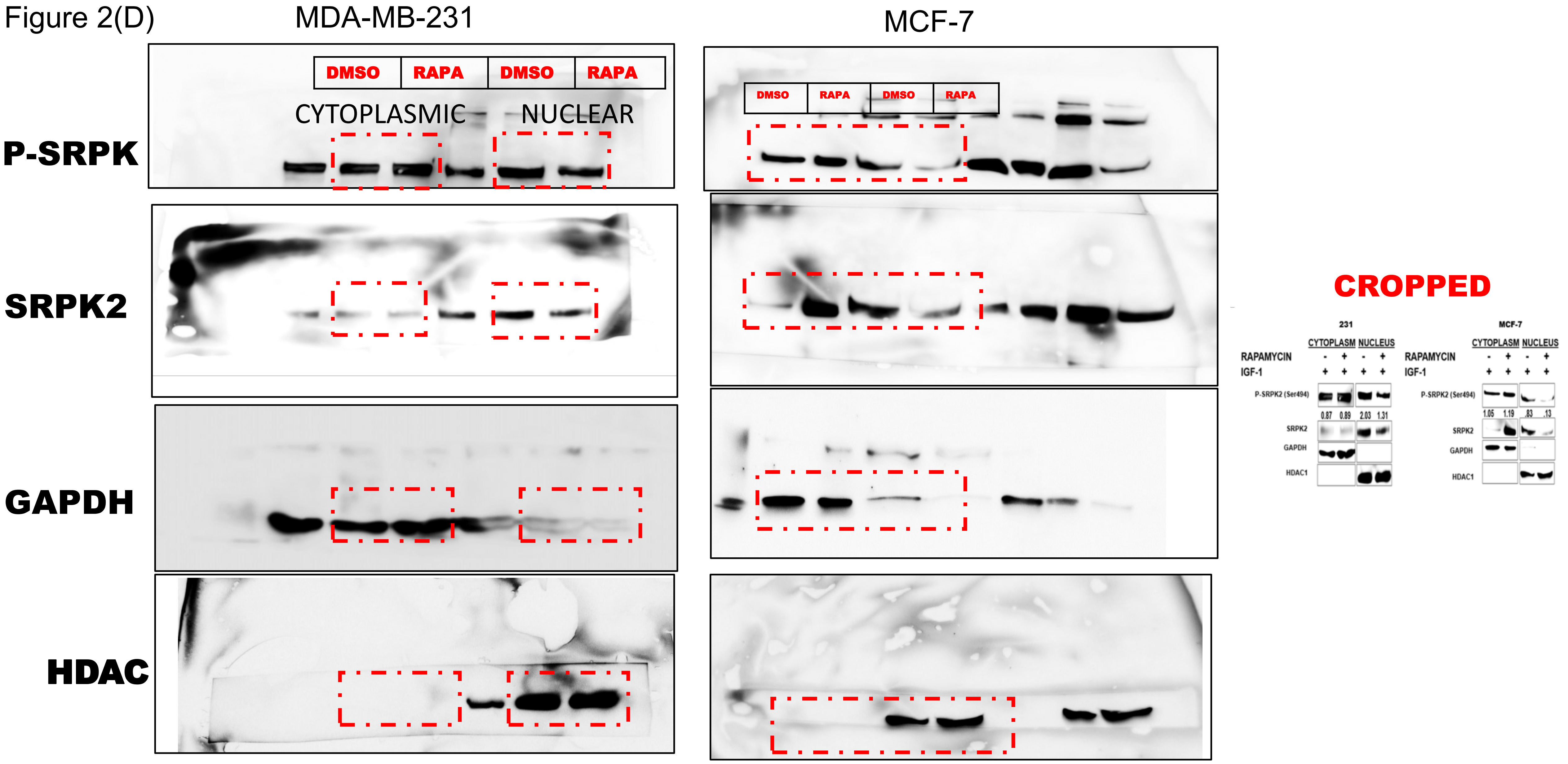
MDA-MB-231
MCF-7
DMSO
RAPA
DMSO
RAPA
CYTOPLASMIC
NUCLEAR
P-SRPK
SRPK2
GAPDH
HDAC
CROPPED
231
MCF-7
CYTOPLASM
NUCLEUS
RAPAMYCIN - + - +
IGF-1 + + + +
P-SRPK2 (Ser494)
0.87 0.89 2.03 1.31
1.05 1.19 .83 .13
SRPK2
GAPDH
HDAC1

Figure 3 (B)

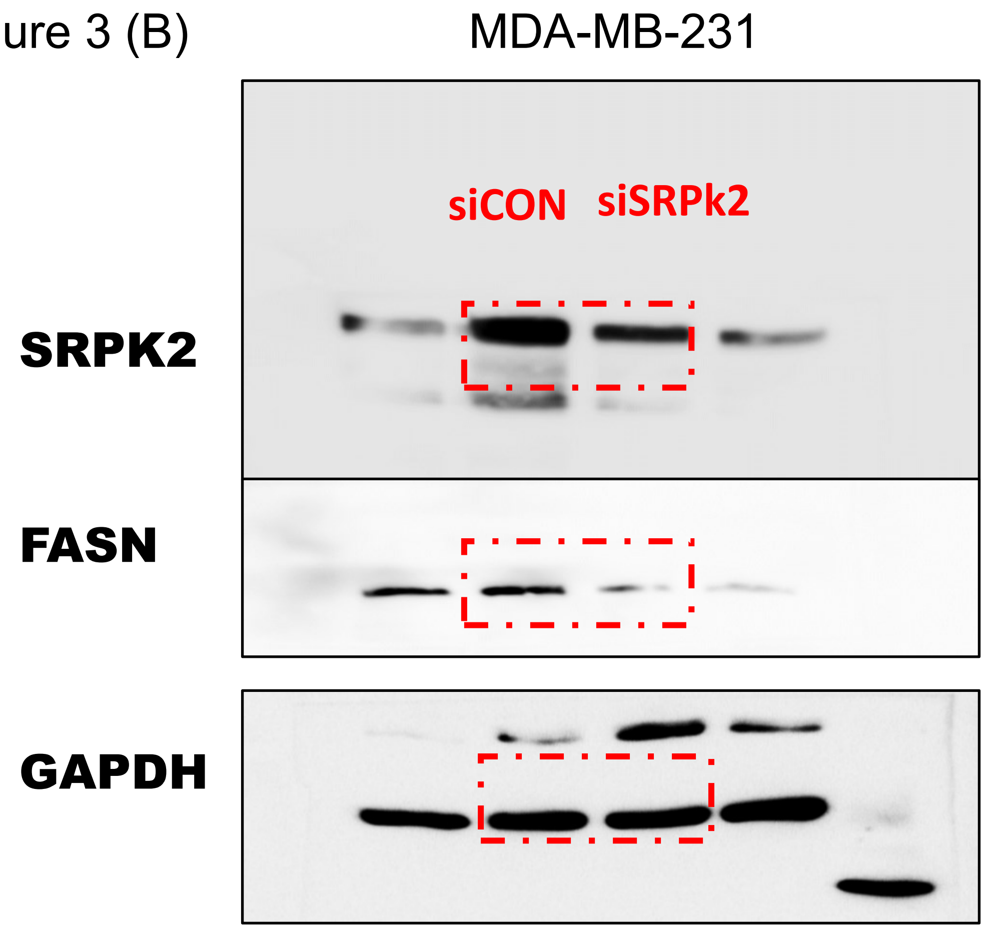

MDA-MB-231
siCON
siSRPk2
SRPK2
FASN
GAPDH

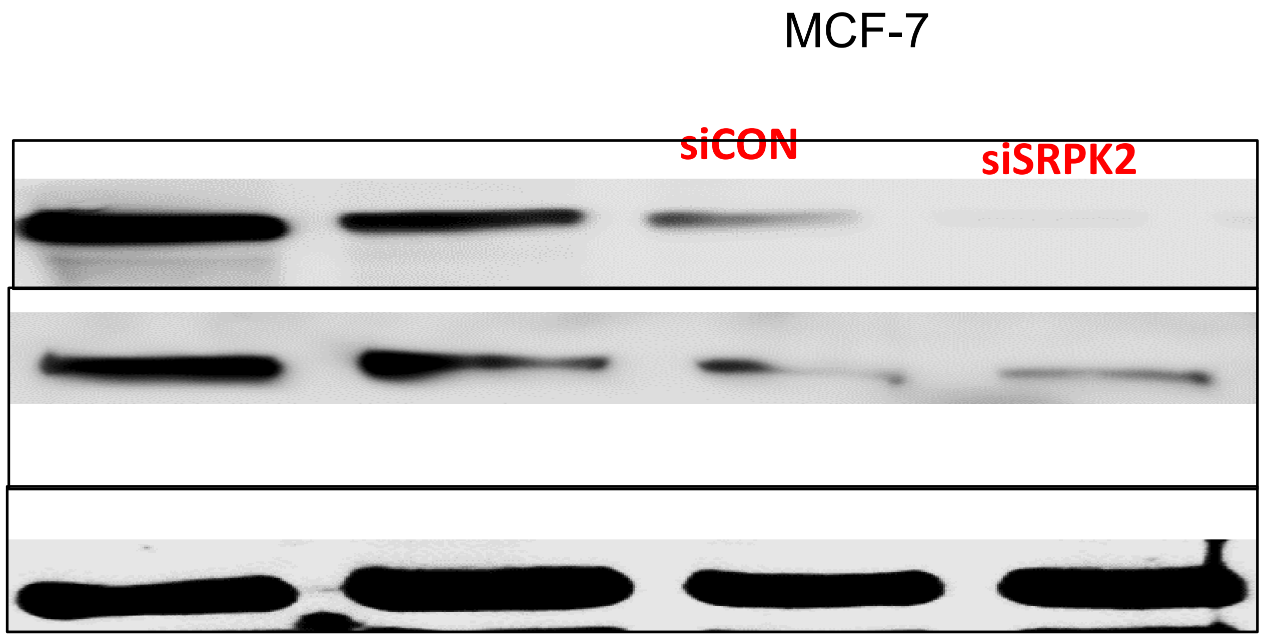

MCF-7
siCON
siSRPK2

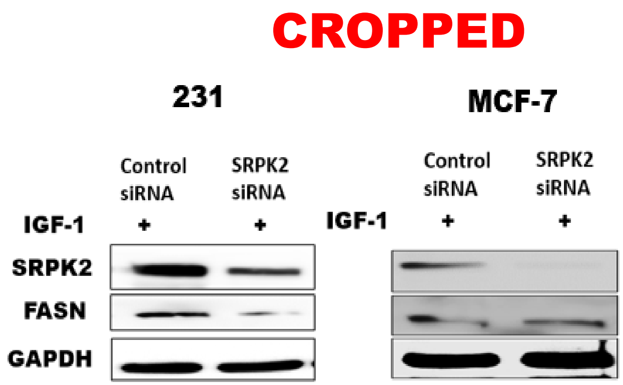

CROPPED
231
Control siRNA
SRPK2 siRNA
IGF-1
+
+
SRPK2
FASN
GAPDH
MCF-7
Control siRNA
SRPK2 siRNA
IGF-1
+
+

Figure 3 (B)

MDA-MB-231

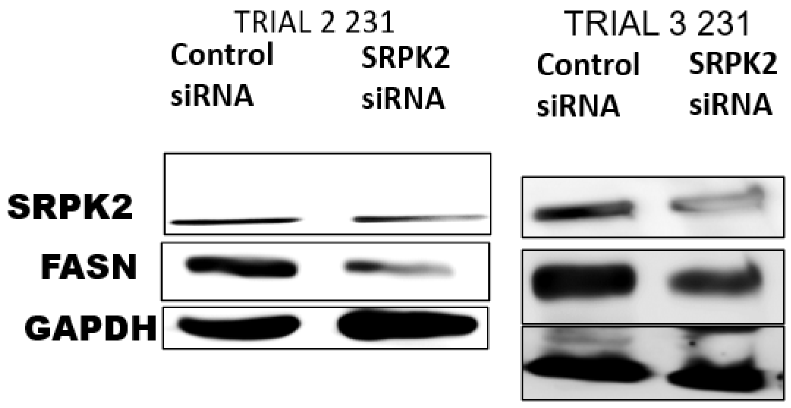

MCF-7 TRIAL 1

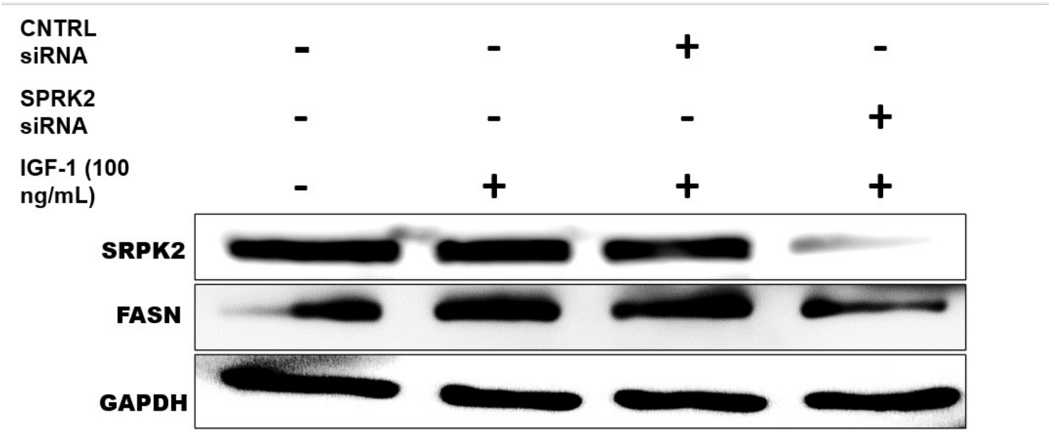

MCF-7 TRIAL 2 AND REPRESENTATIVE IMAGE (TRIAL 3)

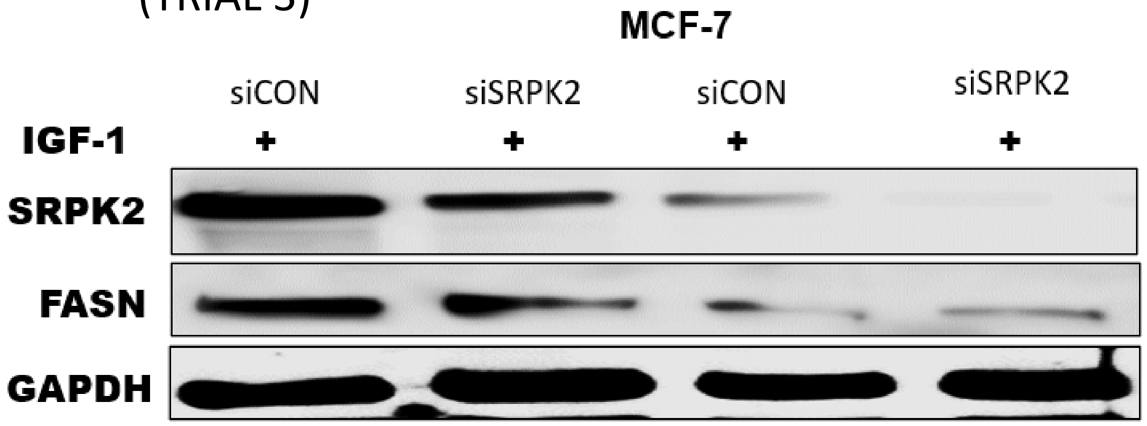

Figure 3 (C)

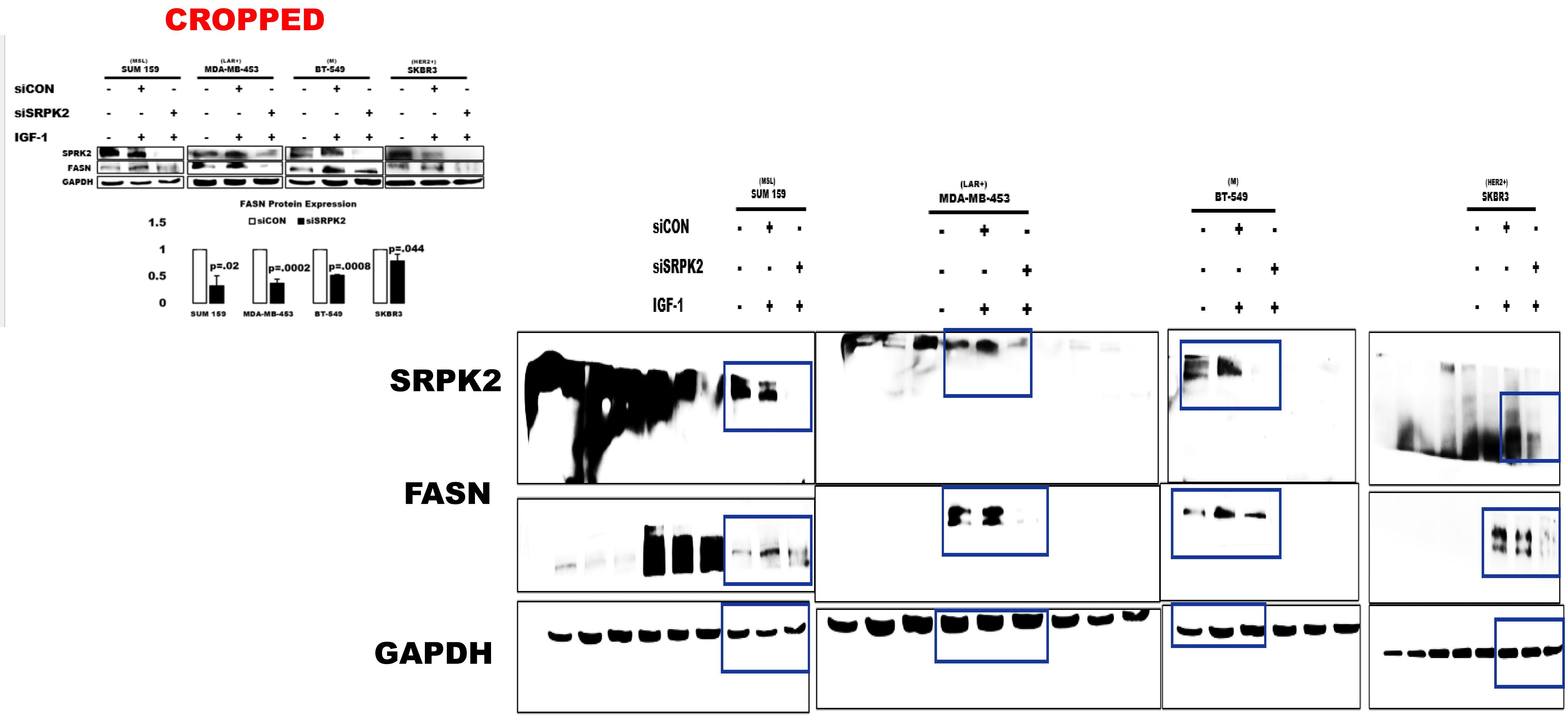
CROPPED
(MSL) SUM 159
(LAR+) MDA-MB-453
(M) BT-549
(HER2+) SKBR3
siCON
siSRPK2
IGF-1
SPRK2
FASN
GAPDH
FASN Protein Expression
siCON
siSRPK2
1.5
1
0.5
0
p=.02
p=.0002
p=.0008
p=.044
SUM 159
MDA-MB-453
BT-549
SKBR3
SRPK2
FASN
GAPDH

SUM 159 REPLICATE 2 & 3

REPLICATE 2

SUM 159

FASN

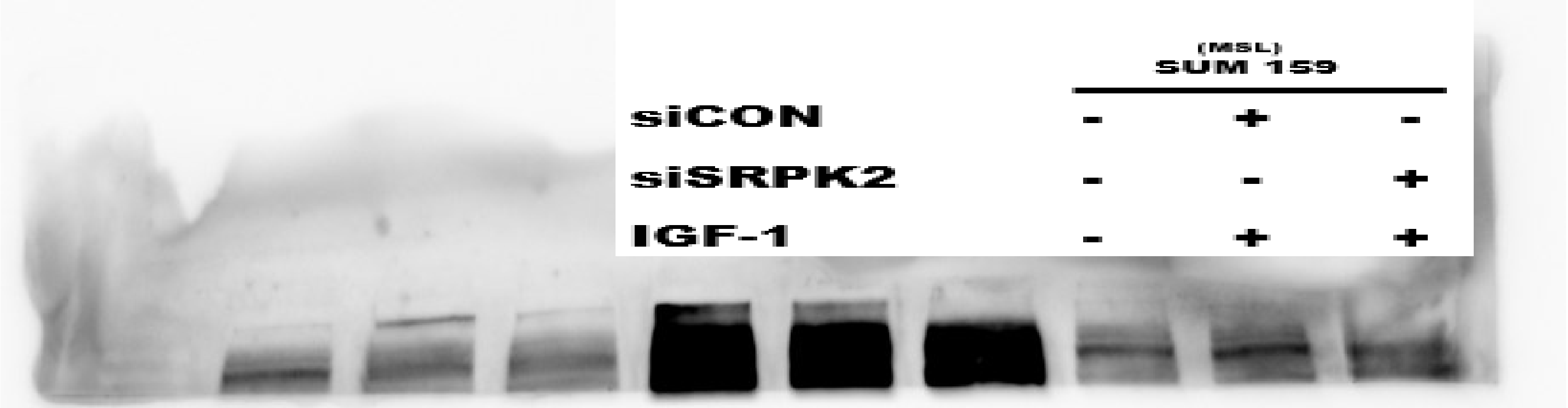

REPLICATE 3

FASN

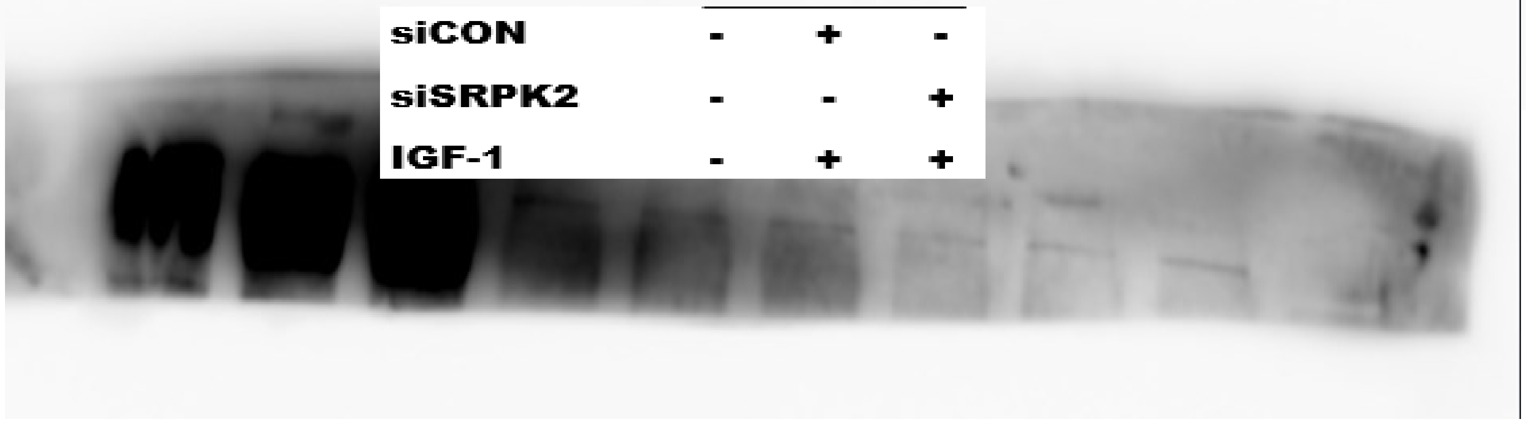

MDA-MB-453 REPLICATE 2 & 3

FASN

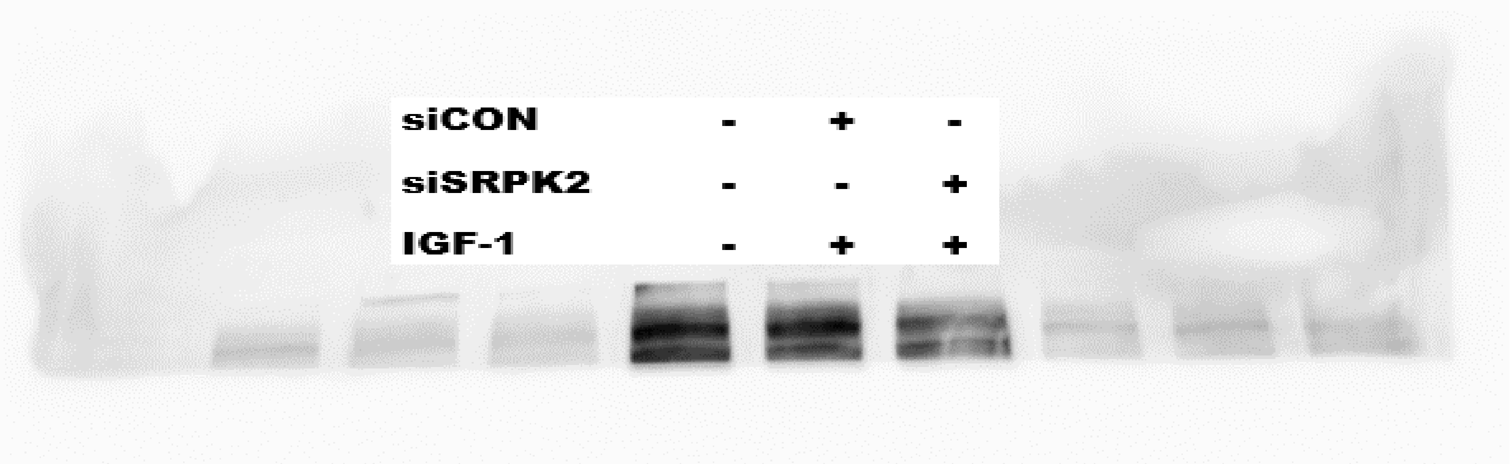

FASN

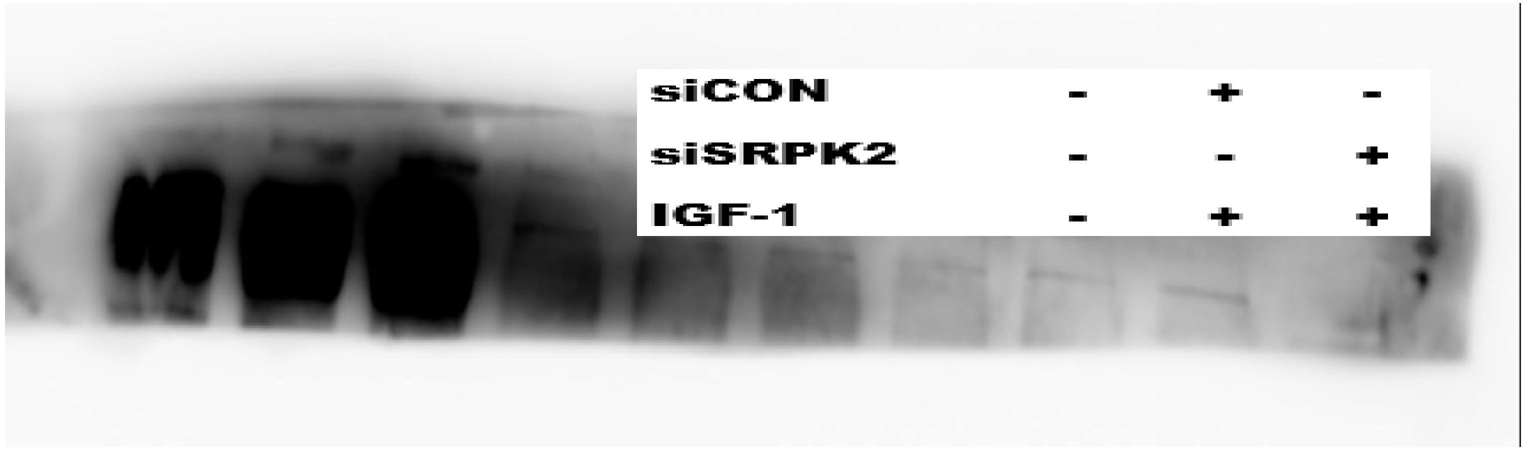

BT-549 REPLICATES 2 & 3

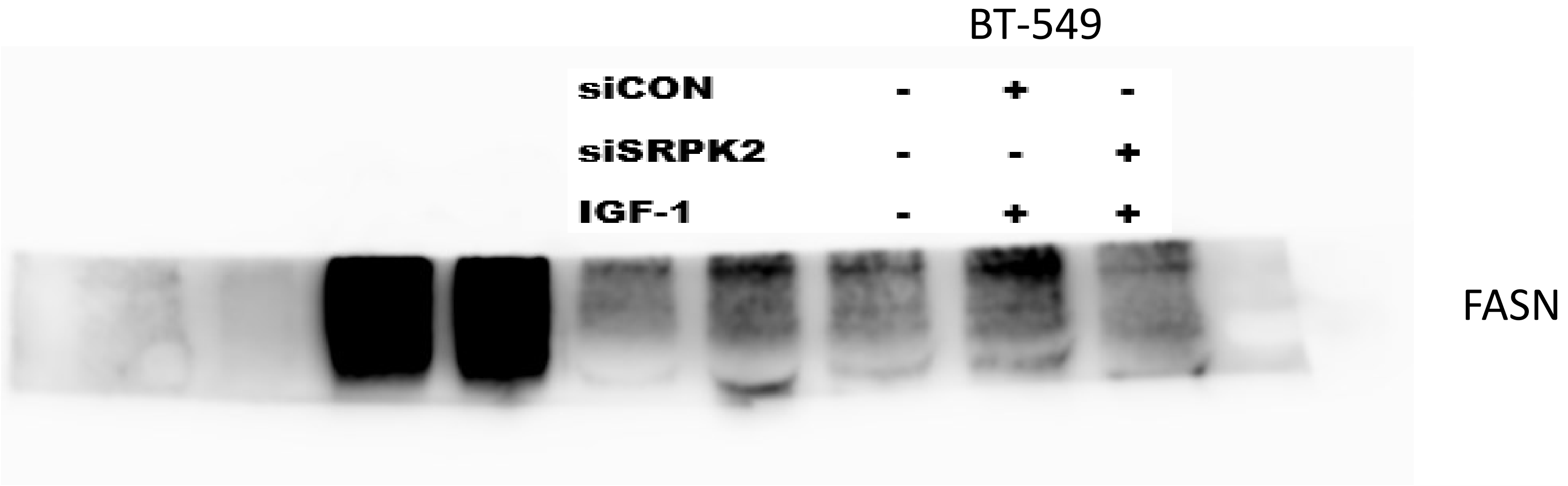

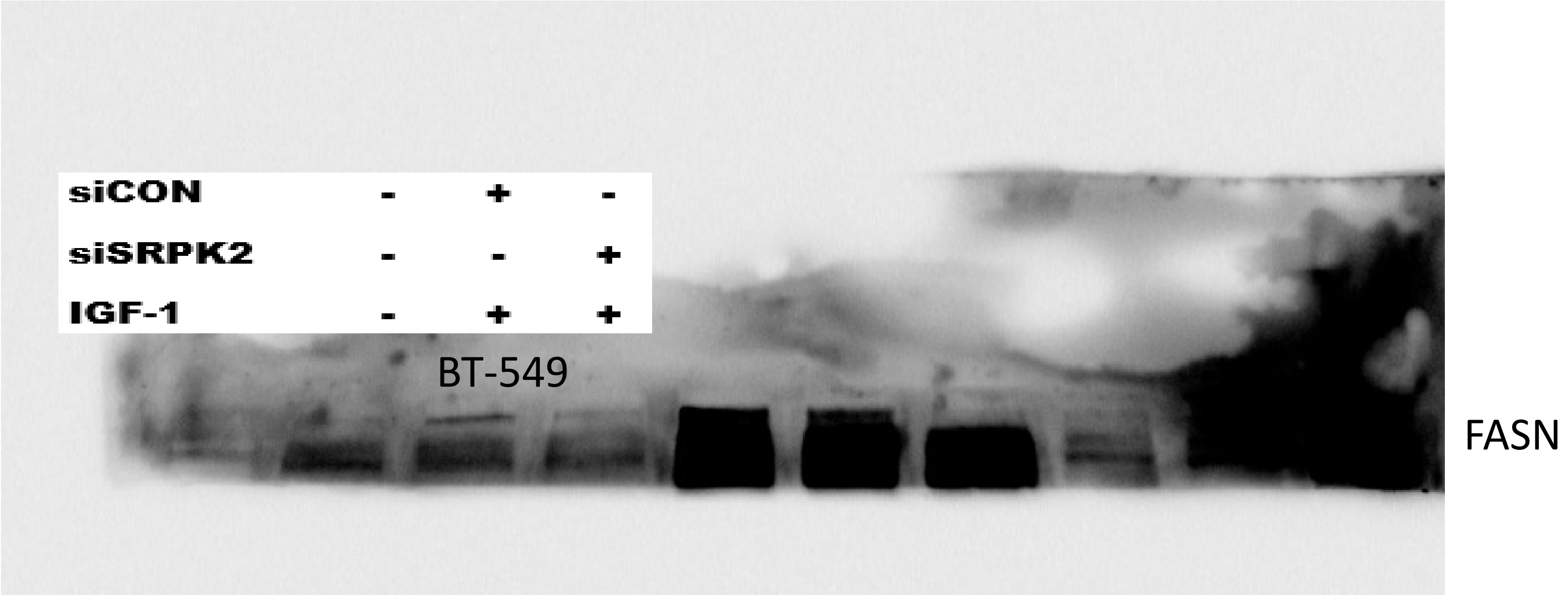

SKBR3 REPLICATES 2 &3

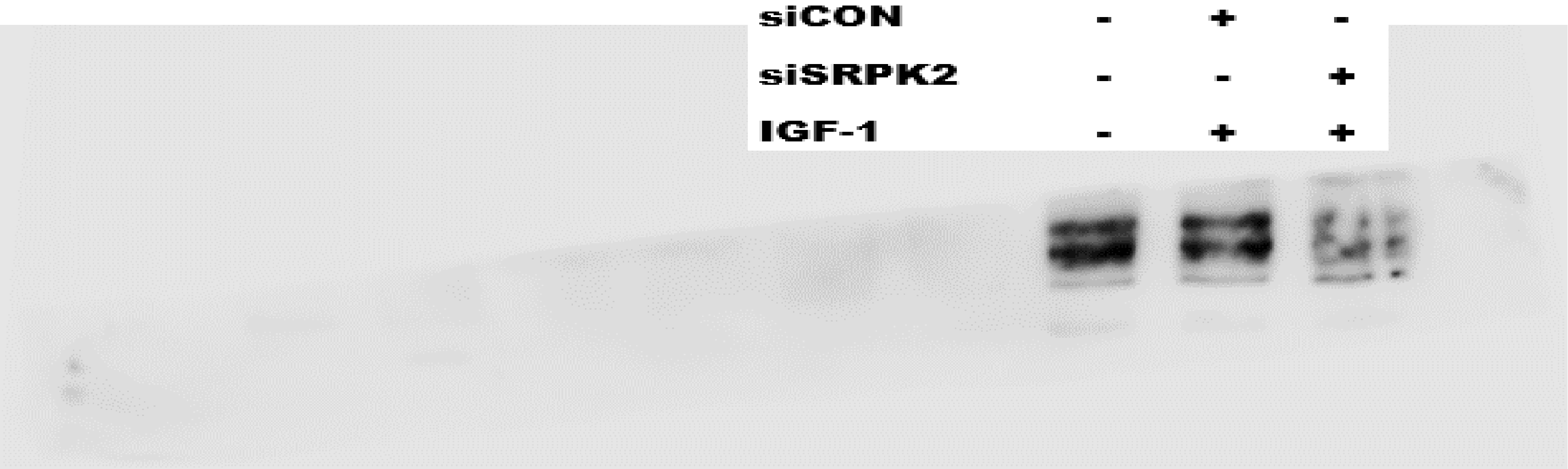

FASN

FASN

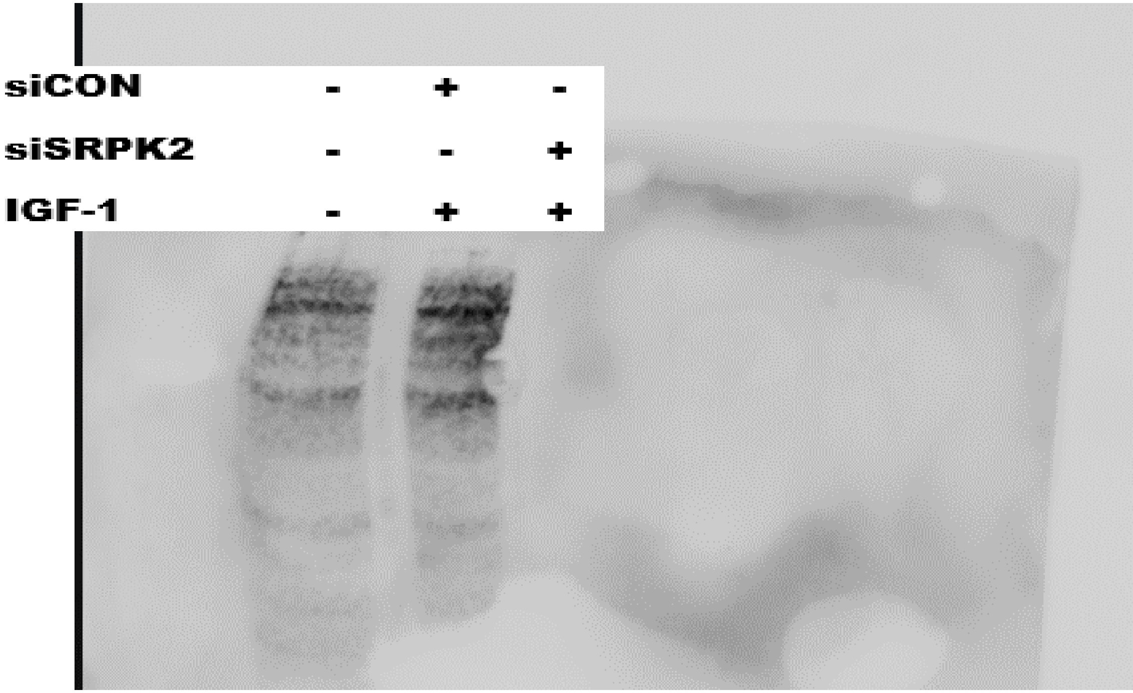

Supplement: Supplementary file 2 — Additional file 2. [file 12885_2022_10062_MOESM2_ESM.pdf]
