# Supplementary material for: An IGF-1R-mTORC1-SRPK2 signaling Axis contributes to FASN regulation in breast cancer
Source: BMC Cancer. 2022 Sep 12;22:976. doi: 10.1186/s12885-022-10062-z (PMC9469522; doi:10.1186/s12885-022-10062-z)
Supplement: Supplementary file 3 — Additional file 3. [file 12885_2022_10062_MOESM3_ESM.pdf]

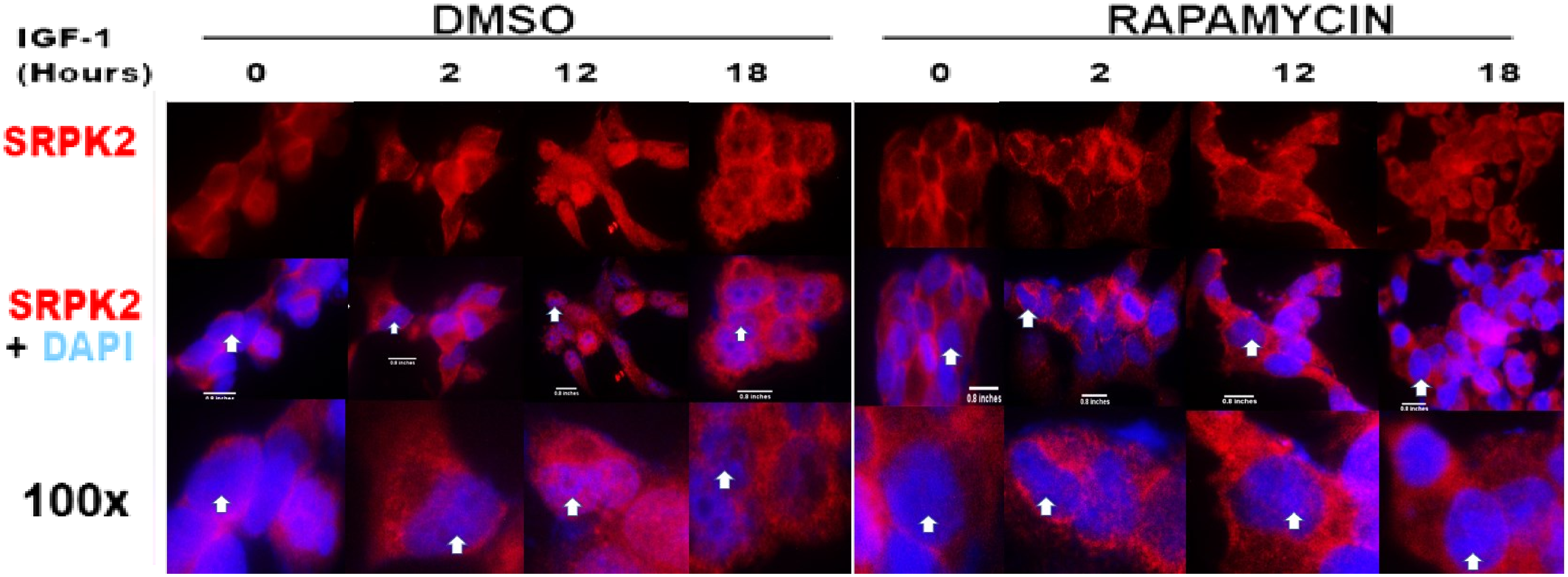

Figure S2. SRPK2 localizes to the nucleus through the IGF-1- mTORC1 pathway , related to figure 2.

A. MCF-7 cells were serum starved 16 hours with either DMSO or rapamycin (100nM) followed by 100ng/mL IGF-1 exposure in full growth medium for 0, 2, 12, and 18 hours. Cells were fixed and stained with anti-SRPK2 antibody followed by secondary mouse Cy3 labelled antibody (Red). Slides were mounted with DAPI to stain nuclei (Blue). Images were obtained on Leica Confocal microscope. Cy3 (SRPK2) and DAPI channels were merged in Image J. Yellow arrows indicate where the image was zoomed in (100X).
